# A Wide Extent of Inter-Strain Diversity in Virulent and Vaccine Strains of Alphaherpesviruses

**DOI:** 10.1371/journal.ppat.1002282

**Published:** 2011-10-13

**Authors:** Moriah L. Szpara, Yolanda R. Tafuri, Lance Parsons, S. Rafi Shamim, Kevin J. Verstrepen, Matthieu Legendre, L. W. Enquist

**Affiliations:** 1 Department of Molecular Biology, Princeton University, Princeton, New Jersey, United States of America; 2 Princeton Neuroscience Institute, Princeton University, Princeton, New Jersey, United States of America; 3 Lewis-Sigler Institute for Integrative Genomics, Princeton University, Princeton, New Jersey, United States of America; 4 VIB lab for Systems Biology and CMPG Lab for Genetics and Genomics, KULeuven, Gaston Geenslaan 1, Leuven, Belgium; 5 Structural & Genomic Information Laboratory (CNRS, UPR2589), Mediterranean Institute of Microbiology, Aix-Marseille Université, Marseille, France; Cornell University, United States of America

## Abstract

Alphaherpesviruses are widespread in the human population, and include herpes simplex virus 1 (HSV-1) and 2, and varicella zoster virus (VZV). These viral pathogens cause epithelial lesions, and then infect the nervous system to cause lifelong latency, reactivation, and spread. A related veterinary herpesvirus, pseudorabies (PRV), causes similar disease in livestock that result in significant economic losses. Vaccines developed for VZV and PRV serve as useful models for the development of an HSV-1 vaccine. We present full genome sequence comparisons of the PRV vaccine strain Bartha, and two virulent PRV isolates, Kaplan and Becker. These genome sequences were determined by high-throughput sequencing and assembly, and present new insights into the attenuation of a mammalian alphaherpesvirus vaccine strain. We find many previously unknown coding differences between PRV Bartha and the virulent strains, including changes to the fusion proteins gH and gB, and over forty other viral proteins. Inter-strain variation in PRV protein sequences is much closer to levels previously observed for HSV-1 than for the highly stable VZV proteome. Almost 20% of the PRV genome contains tandem short sequence repeats (SSRs), a class of nucleic acids motifs whose length-variation has been associated with changes in DNA binding site efficiency, transcriptional regulation, and protein interactions. We find SSRs throughout the herpesvirus family, and provide the first global characterization of SSRs in viruses, both within and between strains. We find SSR length variation between different isolates of PRV and HSV-1, which may provide a new mechanism for phenotypic variation between strains. Finally, we detected a small number of polymorphic bases within each plaque-purified PRV strain, and we characterize the effect of passage and plaque-purification on these polymorphisms. These data add to growing evidence that even plaque-purified stocks of stable DNA viruses exhibit limited sequence heterogeneity, which likely seeds future strain evolution.

## Author Summary

Alphaherpesviruses such as herpes simplex virus (HSV) are ubiquitous in the human population. HSV causes oral and genital lesions, and has co-morbidities in acquisition and spread of human immunodeficiency virus (HIV). The lack of a vaccine for HSV hinders medical progress for both of these infections. A related veterinary alphaherpesvirus, pseudorabies virus (PRV), has long served as a model for HSV vaccine development, because of their similar pathogenesis, neuronal spread, and infectious cycle. We present here the first full genome characterization of a live PRV vaccine strain, Bartha, and reveal a spectrum of unique mutations that are absent from two divergent wild-type PRV strains. These mutations can now be examined individually for their contribution to vaccine strain attenuation and for potential use in HSV vaccine development. These inter-strain comparisons also revealed an abundance of short repetitive elements in the PRV genome, a pattern which is repeated in other herpesvirus genomes and even the unrelated Mimivirus. We provide the first global characterization of repeats in viruses, comparing both their presence and their variation among different viral strains and species. Repetitive elements such as these have been shown to serve as hotspots of variation between individuals or strains of other organisms, generating adaptations or even disease states through changes in length of DNA-binding sites, protein folding motifs, and other structural elements. These data suggest for the first time that similar mechanisms could be widely distributed in viral biology as well.

## Introduction

Alphaherpesviruses are widespread in the human population, with herpes simplex virus 1 (HSV1) and 2 causing oral and genital lesions, respectively, while varicella zoster virus (VZV) causes chicken pox and shingles [1]–[3]. In the agricultural industry, a related veterinary alphaherpesvirus, pseudorabies virus (PRV), causes similar disease in swine and significant economic cost due to weight loss in infected adults and reproductive losses during pregnancy and suckling [4], [5]. As occurs with HSV and VZV, PRV infection has higher morbidity and mortality rates for neonates, with decreasing severity of disease as the age at onset of infection increases [2], [4], [6]. PRV and VZV primarily infect via the respiratory mucosa, while HSV-1 primarily infects at the oral mucosa. VZV infection includes a viremic phase that yields widespread vesicular lesions, while PRV and HSV are usually non-viremic and spread predominantly by mucosal infection and neuronal innervation. These alphaherpesviruses are widespread in the population because of their tendency to infect neurons: they establish lifelong latency in the host peripheral nervous system. These latent neuronal infections may occasionally reactivate and spread back the mucosal surfaces where the infection initiated. After further replication, the viruses can spread to new hosts.

Among alphaherpesviruses, vaccines are available for VZV and PRV, but not HSV [7], [8]. Despite considerable effort and recent progress, no broadly effective vaccine candidates have yet emerged for HSV infection [9]–[11]. The co-morbidities of HSV-1 and HSV-2 with human immunodeficiency virus (HIV), which include increased acquisition of HIV due to the inflammation and lesions caused by HSV infection, have added impetus to the search for a vaccine [10]–[13]. PRV serves as a useful model for HSV pathogenesis and vaccine development, because of their similar infectious cycle and ability to infect a variety of animal models [4], [5], [8], [14]–[17]. In contrast, VZV has a more restricted tropism for human cells that complicates its study in animal models [18]–[20]. The agricultural importance of PRV and relative ease of vaccine testing has led to the development of several PRV vaccine strains, whose genetic characteristics have been determined by mapping isolated genomic fragments and sequencing of select regions [8], [21]–[23]. Of note, the vaccine strain Bartha has a well-characterized deletion of several viral proteins that attenuates its virulence and also limits its spread in neurons, which led to its subsequent development as a tool for trans-neuronal tracing [21], [24]–[27]. Like several other early vaccine strains, PRV Bartha was attenuated by extensive passage in the laboratory, thus making the full discovery of its genome-wide mutations a priority [22], [23], [28], [29]. Because the only available PRV genome sequence to date is a mosaic of six strains [30], it has been difficult to discern whether mutations detected in PRV Bartha and other vaccine strains are unique or represent ordinary sequence diversity, *i.e.* are found in other wild-type genomes [31]–[35]. We therefore applied our recent success in using Illumina high-throughput sequencing (HTS) to obtain HSV-1 strain genomes to determining the sequence diversity in the PRV vaccine strain Bartha.

In addition to sequence polymorphisms, insertions, and deletions, another major class of variation between nucleic acid sequences lies in copy number variation, either of coding sequences or of repeated structural elements. Herpesvirus genomes have long been known to contain several sites with tandem short sequence repeats (SSRs) or reiterations [36]–[40]. Variation in these elements has been described both within and between herpesvirus strains, but their functions were largely unexplored [22], [35], [41]–[43]. SSRs can be transcription factor binding sites, chromatin insulators, protein folding motifs, or other regulatory elements [44], [45]. Recent studies have shown that SSR expansion and contraction, most likely through recombination or polymerase slippage, can generate phenotypic variation [46]–[49]. A range of human diseases result from SSR expansion or contraction, including the transcriptional silencing of the gene FMR1 via an upstream SSR, which causes Fragile X syndrome, and the poly-glutamine tract expansion in huntingtin protein, which causes Huntington's disease [50]–[53]. Limited explorations of repetitive elements in viral genomes suggest that SSRs in viral genomes likewise play functional roles [54]–[57]. To explore SSR prevalence and function in herpesviruses, we initiated a global SSR assessment and comparison across viral species, as was recently done for a variety of fungal and bacterial pathogens [49], [58]. These data highlight the contribution of SSRs to overall sequence diversity in viruses, and through the presence of these elements in both coding and non-coding regions, suggest that viral SSRs may likewise have the potential to affect gene expression and protein functions.

We sequenced three widely-studied PRV isolates by HTS: the attenuated vaccine strain Bartha and the virulent strains Kaplan and Becker. This analysis reveals genome-wide sequence diversity between strains, both in the PRV proteome and also in many SSRs. Our comparison of protein coding sequences revealed that 46 of 67 PRV proteins have changes in the vaccine strain Bartha which are not found in the virulent Kaplan or Becker strains. We mapped homologous SSRs in all three strains and provide a comprehensive overview of inter-strain variation in SSR length. We compared the proportion of SSRs in PRV to those found in HSV-1, VZV, the human betaherpesvirus cytomegalovirus (HCMV) and gammaherpesviruses Epstein-Barr virus (EBV) and Kaposi's sarcoma-associated herpesvirus (KSHV), and Mimivirus. We find that SSRs are likely to be a common property of these large DNA viruses. Finally, we examined the limited number of polymorphic bases detected in these plaque-purified virus stocks, and tested the rate of polymorphism occurrence in purified and non-purified virus populations. These data on sequence variation in PRV strains expand our understanding of viral genome diversity and how attenuated strains lead to successful anti-viral vaccines.

## Results/Discussion

### Sequencing and assembly of multiple PRV strain genomes

**Figure 1:**
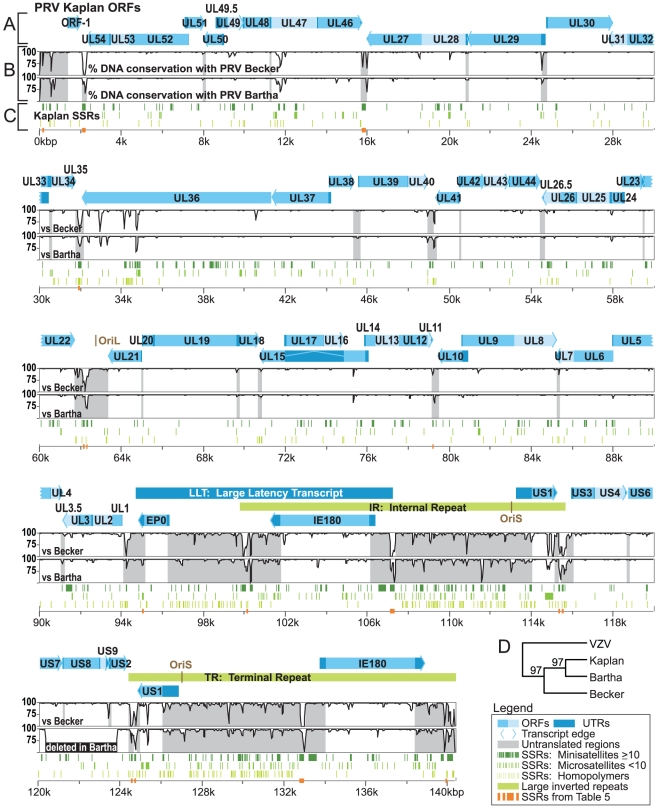
Genome organization of PRV Kaplan and comparison of sequence conservation with strains Becker and Bartha. A) Open reading frames (ORFs) are plotted spatially along the genome, along with their untranslated regions (UTRs). The genome proceeds from the Unique Long (UL) region into the Unique Short (US) region, with US being flanked by long inverted repeats (IR, TR). The large-latency transcript (LLT) is not translated. A horizontal bar connects the spliced portions of the UL15 ORF; splicing in the 5′ UTR of US1 is not shown for space reasons. B) Graph depicts DNA sequence conservation between PRV Kaplan and PRV Becker or PRV Bartha. Conservation is calculated from a multiple sequence alignment, and the conservation score between any two genomes is plotted from a sliding 100 bp window. C) Short sequence repeats (SSRs) are plotted as they occur along the PRV Kaplan genome. SSRs include minisatellites (repeat unit ≥10 bp), microsatellites (repeat unit <10 bp), and homopolymers (minimum length 6). D) A phylogenetic tree based on a whole-genome multiple sequence alignment demonstrates the closer relationship of PRV strains Kaplan and Bartha. Bootstrap values are shown at branch points (see Methods for details). The same result was obtained using nucleotide sequences of gC (data not shown), as was done for several recent PRV phylogenetic comparisons [33], [34], [177].

We used Illumina deep sequencing and bioinformatic analyses to assemble millions of sequence reads into three completed genomes of PRV Kaplan, Becker, and Bartha. To produce genetically homogeneous stocks for sequencing, we purified a single plaque from each virus stock, plated it out, selected a progeny plaque, and repeated the process. These plaque-purified stocks were then used to produce viral nucleocapsid DNA for Illumina genomic DNA libraries. Over 15 million Illumina sequence reads were combined for each strain (details of HTS sequence reads for each strain are listed in Table S1 in Text S1). High quality viral sequence data were used for a 3-phase *de novo* assembly process (see Methods for details): 1) the automated generation of large blocks of continuous sequence, or contigs, from Illumina sequence data (usually 0.1–30 kilobase pairs (kb) in length), 2) the automated generation of super-contigs (1–60 kb) using a long-read assembler, and 3) the manual curation of gaps, joins, and annotations. Assembly quality was checked by BLAST-based alignment of each new genome versus the prior mosaic reference. PCR-validation confirmed regions of the assembly with greatest divergence from the mosaic strain, and guided genome correction in selected regions of the assembly (Figure S1 and Table S2 in Text S1). The resulting genomes resembled the original mosaic genome in overall size and gene content (Figure 1A). The PRV genome is organized into a unique long (UL) region and a unique short (US) region, with large inverted and terminal repeats (IR, TR) flanking the US region. Overall, DNA sequences are largely conserved between PRV Kaplan, Becker, and Bartha, with the greatest foci of divergence occurring in IR/TR and noncoding regions (Figure 1B). Phylogenetic comparison of the three full-length genomes revealed a closer relationship between PRV strains Kaplan and Bartha than PRV Becker (Figure 1D).

**Figure 2:**
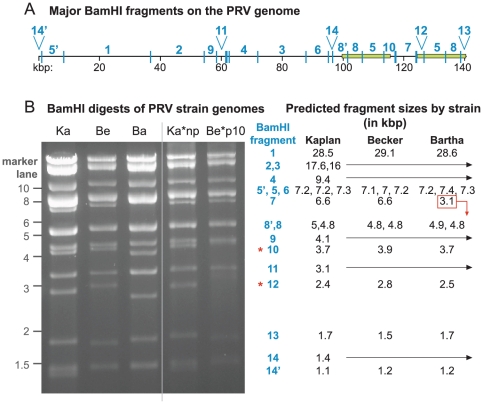
BamHI RFLP confirmation of PRV genome assemblies. A) Location of major BamHI fragments along the PRV Kaplan genome. Fragments are identified by historical fragment numbering [41]. Genome position in kilobase pairs (kb) is listed below the fragments, with the large inverted repeats IR and TR shown as green boxes. B) RFLP analysis of BamHI fragments of PRV strains Kaplan (Ka), Becker (Be), Bartha (Ba), an unpurified Kaplan stock (Ka*np), and a Becker stock passaged 10 times *in vitro* (Be*p10). Positions of a standard marker are noted on the left. Major BamHI fragments, and their predicted size in each strain, are indicated to the right at their approximate height in the gel image. The Bartha deletion causes a major size shift in BamHI fragment 7 (boxed; now 3.1 kb); the new band co-migrates with BamHI fragment 11 (also 3.1 kb). All bands except the variable fragments 10 and 12 (red asterisks) match their predicted sizes. Arrows from Kaplan through Becker and Bartha columns indicate bands that are predicted to be of equivalent size in all three strains.

To ascertain the quality and depth of coverage of these new genomes, sequence reads were aligned back to the assembled genomes. Median coverage was very high: 3,704 sequence reads per base for PRV Kaplan, 4,145 reads/base for Becker, and 4,137 reads/base for Bartha (see also Table S1 in Text S1). This coverage was reduced in genome regions with extremely high or low G/C content, as has been observed for both eukaryotic and bacterial genomes (Figure S2A,B in Text S1) [59], [60]. In addition to analyzing coverage depth, the resulting genomes were used to predict restriction digest patterns, which were compared to actual restriction fragment length polymorphism (RFLP) patterns (Figure 2). Digest patterns match the predicted fragment sizes, with the exception of two classically variable fragments (BamHI 10 and 12; Figure 2) that have been observed to differ even between repeated passages of the same strain [22], [41], [42].

### Genetic differences and pathogenicity in the vaccine strain PRV Bartha

**Figure 3:**
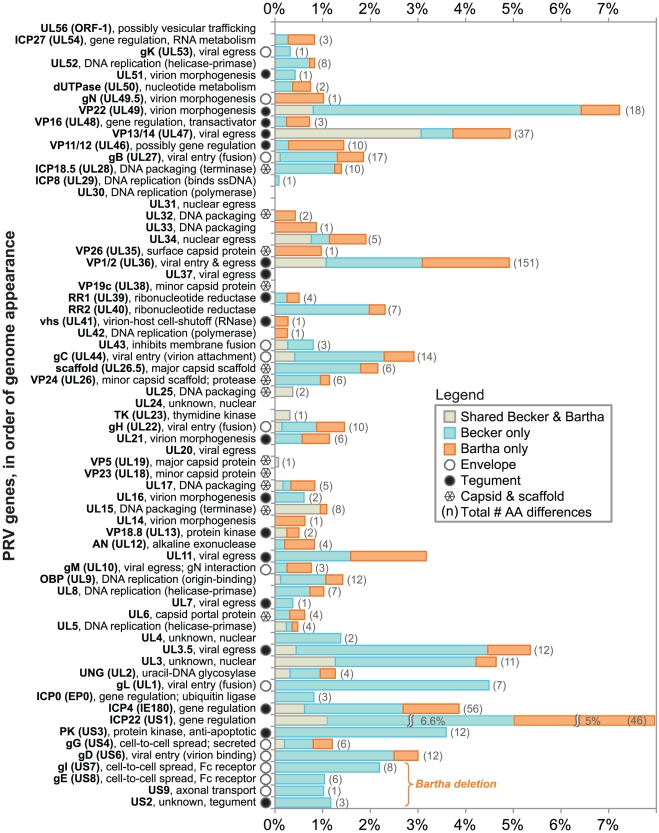
Protein coding variation in PRV Bartha and Becker, vs. the reference strain Kaplan. The percent of AAs differing in PRV Becker and Bartha, vs. the new reference genome of PRV Kaplan, are plotted in order of occurrence along the PRV genome (top to bottom). Eight proteins show no inter-strain variation in coding sequence. The total number of differences (Tables 1–3) have been normalized to protein length. Protein names and functions are listed on the left, along with a symbol indicating if the protein product is a known virion component. AA differences from the reference strain Kaplan are categorized as being unique to the vaccine strain PRV Bartha (orange), unique to the virulent strain PRV Becker (blue), or shared (observed in both Bartha and Becker; gray). The four proteins affected by the deletion in Bartha's US region are bracketed at the bottom.

PRV Bartha displays the most divergent phenotype of the PRV strains sequenced here, with severe attenuation of virulence *in vivo* conferring its suitability for use as a vaccine strain. We compared all protein coding regions of PRV Bartha and the two wild-type strains PRV Kaplan and Becker, to search for novel sequence differences corresponding to potential effects on pathogenicity and attenuation of the vaccine strain (Tables 1–3). Prior studies mapped a deletion in the Bartha US region that removes all of gE (US8) and US9 and creates an fusion of gI (US7) and US2, as well as subtle variations in gC (UL44), gM (UL10), and UL21 [21], [28], [31], [61]–[65]. Our *de novo* assembled Bartha genome confirms the boundary of the US region deletion (position 120,927 on the Bartha genome) as originally mapped by Maxam-Gilbert sequencing [66]; this region spans 3,482 bases on the reference PRV Kaplan genome (positions 120,363–123,845; see also Figure 1B). Adding to these previously reported findings, we identified a total of 46 proteins with coding differences that are unique to PRV Bartha and not found in either wild-type strain (Table 1 and Figure 3). Several of these amino acid (AA) changes are conservative, such as a minor Ala13Val change in Bartha's VP18.8 (UL13), or represent expansions or contractions associated with AA repeats (*e.g.* VP1/2/UL36, ICP4/IE180, AN/UL12). Many mutations affect loosely mapped functional protein domains, for instance two differences in the 300 AA chemokine-binding domain of Bartha's gG [67]. Further studies will be necessary to define any functional effects in these regions.

**Table 1:** Protein-coding variations unique to the vaccine strain PRV Bartha, in comparison to the virulent strains PRV Becker and Kaplan.

Gene a	Amino acid residues found in PRV Bartha, which differ from PRV Becker & Kaplan b
**ICP27 (UL54)**	G9D, T258A
**UL52**	A824V
**dUTPase (UL50)**	S20G
**gN (UL49.5)**	**L7P**
**VP22 (UL49)**	T126A, 129(+A)
**VP16 (UL48)**	Q171R, T386A
**VP13/14 (UL47)**	V7A, E63D, E84G, 96–99(EEEE>Δ), Y144H, P432A
**VP11/12 (UL46)**	W108R, 301–304(AAVV>Δ), 508–509(GE>Δ), A532T
**gB (UL27)**	Y267H, R440Q, **506–507(SP>AA), P509Q**
**ICP18.5 (UL28)**	A517V
**UL32**	W266R, A289V
**UL33**	7(+G)
**UL34**	D188G, Y221C
**VP26 (UL35)**	H65R
**VP1/2 (UL36)** c	T811A, L2152P, **2180(+EPTPAAKLAPPAPPPAKPA** * **)_2_,** L2182T, H2219P, P2314L, K2449R, V2465A, T2666A, 2686(+PGDDVVPA), V2853F, E2876G
**RR1 (UL39)**	S95N, T294A
**RR2 (UL40)**	G266D
**vhs (UL41)**	M353V
**UL42**	E331A
**gC (UL44)**	L14P, E43A, P156S
**scaffold (UL26.5)**	120(+Q)
**VP24 (UL26)**	365(+Q)
**gH (UL22)**	V59D, **P438S**, 481–482(EE>G)
**UL21**	H37R, E355D, V375A
**UL17**	G239D, S341G, P521L
**UL15**	K404R
**UL14**	Q18R
**V18.8 (UL13)**	**A13V**
**AN (UL12)**	**178(+GD),** V474A
**UL11**	Y49C
**gM (UL10)**	59–60(TS>AP)
**OBP (UL9)**	D4P, 6–7(GG>RV)
**UL8**	A52V, V509I
**UL6**	4(+AA)
**UL5**	A2Δ
**UL3.5**	H94Q, R193H
**UL3**	T97Δ
**UNG (UL2)**	A286S
**ICP4 (IE180)**	**Q92Δ,** R195Δ, S234P, N296S, L319P, 371(P>LA), S752A, 854(+ESGSST), S954Δ, V1218A, H1390R
**ICP22 (US1)**	V31L, 239(+DEDEEEEE), 254(+DEDGLCEDE)
**gG (US4)**	**306–307(RG>HR)**
**gD (US6)**	V69A, N82S
**gI (US7), gE (US8), US9, US2**	*Bartha deletion region*

a Proteins are listed in order of occurrence along the genome (see Figure 3 for full list and functions). Table 2 lists proteins with AA differences from PRV Kaplan that are shared by PRV Bartha and Becker strains.

b Bold indicates AA differences discussed in the text. Single AA residues changes are written in standard format, including the Kaplan reference strain AA, its position, and the AA residue found in Bartha's protein sequence, *e.g.* S100P. Insertions in PRV Bartha are indicated by the AA position in Kaplan followed by “+” and the new AAs, *e.g.* 100(+RR). Deletions are indicated by the symbol Δ. Sequential changes are combined and shown with the Kaplan strain AA positions first, followed by the relevant Kaplan AA residues, then “>”, and finally the new alternative AA residues, *e.g.* 100–102(RAR>EDA).

c Subscript on parentheses in UL36 indicates multiple copies of a repeating unit.

*Final alanine (A) in this repeat unit changes to valine (V) in the second copy of the repeat unit.

**Table 2 ppat-1002282-t002:** Protein-coding variations shared by the vaccine strain PRV Bartha and the virulent strain PRV Becker, in comparison to the reference strain PRV Kaplan.

Gene a	Amino acid residues found in PRV Bartha and Becker, which differ from PRV Kaplan b
**VP22 (UL49)**	G63D, A109T
**VP13/14 (UL47)**	T75M, D115E, E124D, D126Δ, 129–130(GD>EE), 133–134(GD>EE), D140G, 151–158(ASRAAAGP>V), S176P, 230–233(STAA>Δ), T658A
**gB (UL27)**	T556A
**UL34**	M172L, G181Δ
**VP1/2 (UL36)** c	G184S, G475E, G1886–1887(GA>AP), A2110T, A2468T, 2782(+PAPPPSR)_3_, L2886P, L2983P, 2993–2996(GDED>Δ)
**UL43**	A299T
**gC (UL44)**	E99K, A181V
**UL25**	S36G, V231A
**TK (UL23)**	V284A
**gH (UL22)**	I539L
**VP5 (UL19)**	P502A
**UL17**	R31A
**UL15**	A158Δ, 203(+RGRGGG)
**V18.8 (UL13)**	V233F
**OBP (UL9)**	T233A
**UL5**	A638P, M661V
**UL3.5**	Q115Δ
**UL3**	R42C, L61R, W104R
**UNG (UL2)**	P82A
**ICP4 (IE180)**	69(+EA), T305P, S353G, 383–385(SSS>Δ), R747Q, S859G
**ICP22 (US1)**	299–300(ED>Δ), D302E, G322E
**gG (US4)**	G348A

a Proteins are listed in order of occurrence along the genome (see Figure 3 for full list and functions).

b Single AA residues changes are written in standard format, including the Kaplan reference strain AA, its position, and the AA residue found in the alternative protein sequence, *e.g.* S100P. Insertions are indicated by the AA position in Kaplan followed by “+” and the new AAs, *e.g.* 100(+RR). Deletions are indicated by the symbol Δ. Sequential changes are combined and shown with the Kaplan strain AA positions first, followed by the relevant Kaplan AA residues, then “>”, and finally the new alternative AA residues, *e.g.* 100–102(RAR>EDA).

c Subscript on parentheses in UL36 indicates multiple copies of a repeating unit.

**Table 3:** Protein-coding variations unique to the virulent strain PRV Becker, in comparison to strains PRV Bartha and Kaplan.

Gene a	Amino acid residues found in PRV Becker, which differ from PRV Bartha & Kaplan b
**ICP27 (UL54)**	H126R
**gK (UL53)**	D78G
**UL52**	H208R, A512V, S600P, D606G, 666–667(LV>PA), P678A
**UL51**	S138N
**dUTPase (UL50)**	L110P
**VP22 (UL49)**	T28A, 30–31(TT>AA), 34(+VPT), 47–49(YDD>Δ), R74H, 123–126(TTTT>A)
**VP16 (UL48)**	G55S
**VP13/14 (UL47)**	89–91(GDE>ADGD), 144–145(YD>RG)
**VP11/12 (UL46)**	A236P, D637A
**gB (UL27)**	72–74(VPG>Δ), S75G, P76T, L78A, T96S, N445S, S496A, S682N, F709L
**ICP18.5 (UL28)**	M58T, R225H, 252(+ASTAAA), A688G
**ICP8 (UL29)**	A1146V
**UL34**	G185Δ
**VP1/2 (UL36)^c^**	A249G, 266–272(AAAPAPA>Δ), P281S, P1083S, 2169(+APAAPAAPPPAKPAEPTPAAKL), L2182P, T2324A, 2326(+TAAT), T2334N, 2404–2417(PPSAQTTLPRPAPP), L2475F, T2535A, 2669(+A), 2762-2763(AA>VV), A2767T, T2772S, A2785P, V2853L
**RR1 (UL39)**	D358G, A376T
**RR2 (UL40)**	G107N, E123G, E138A, A140E, V142I, D209S
**UL43**	L239F, V311A
**gC (UL44)**	G30D, 68–69(RA>PV), T179A, 183–184(ED>V), G187E, D317A, R477Q
**scaffold (UL26.5)**	129(+HP), A203Δ, 244(+AP)
**VP24 (UL26)**	375(+HP), A449Δ, 490(+AP)
**gH (UL22)**	L58P, S201A, E478G, 480(+EE)
**UL21**	347–349(DDP>L)
**UL17**	E178K
**UL16**	A120G, P121A
**AN (UL12)**	R124H
**UL11**	A62V
**gM (UL10)**	A292T
**OBP (UL9)**	255–257(AGA>Δ), S266G, A281T, 284–285(TA>AV), E653G
**UL8**	A3T, G125Δ, E138G, L331F, I647V
**UL7**	R148Q
**UL6**	E183D, A463G
**UL5**	P580Δ
**UL4**	S21P, V118A
**UL3.5**	A38T, P53H, Q95H, 113–114(QQ>Δ), G132D, H189N, A199T, A201T
**UL3**	P50R, D69G, T97A, 153–155(ARR>Δ), P159A
**UNG (UL2)**	A27Δ, S55P
**gL (UL1)**	T20P, H27R, P35A, A45H, G62D, T84N, I122V
**ICP0 (EP0)**	Q33L, S34T, A172T
**ICP4 (IE180)**	P182S, P229S, N294S, T298A, P327L, 371(+AA), 436(+AAS), A792G, S851G, A909S, 947(+SSS), E991A, G1195E, T1355A, V1359L, L1369R, A1393S, S1396R, A1398P, 1400–1402(AGP>ED), 1406–1408(GDS>SAA), V1414F
**ICP22 (US1)**	1(+DR), 4(V>A), R84H, E237A, 244–260(GETDVYEEDDEAEDEED>Δ), 333(+ED)
**PK (US3)**	27(+GD), A45S, V48A, Q97K, S100A, 106–107(RM>SL), A116V, C146R, A150S, T242A
**gG (US4)**	A244V, L249S, T484I
**gD (US6)**	I122V, N207S, E209D, R212K, 266(+PR), V284A, G290D, H305R, T391A
**gI (US7)**	Q207P, L210I, A249V, V255A, D257G, 260(+L), S281G, S337N
**gE (US8)**	T124M, R162Q, Q180R, T299A, G446A, V499D
**US9**	P24A
**US2**	L130I, V146I, T182A

a Proteins are listed in order of occurrence along the genome (see Figure 3 for full list and functions).

b Single AA residues changes are written in standard format, including the Kaplan reference strain AA, its position, and the AA residue found in the PRV Becker protein sequence, *e.g.* S100P. Insertions are indicated by the AA position in Kaplan followed by “+” and the new AAs, *e.g.* 100(+RR). Deletions are indicated by the symbol Δ. Sequential changes are combined and shown with the Kaplan strain AA positions first, followed by the relevant Kaplan AA residues, then “>”, and finally the new alternative AA residues, *e.g.* 100–102(RAR>EDA).

**Figure 4:**
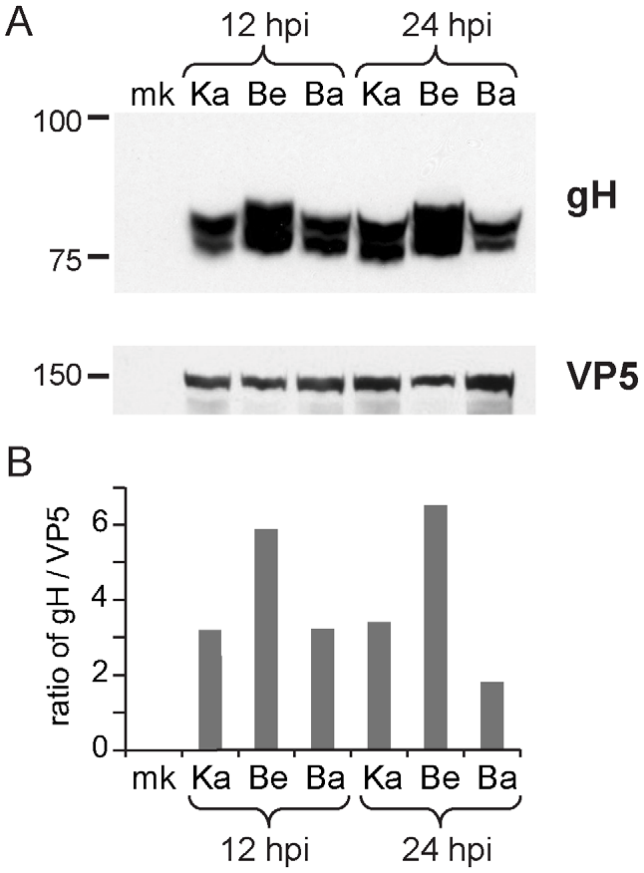
Inter-strain variation in protein levels of gH. A) Western blot analysis of infected cell lysates demonstrates that PRV Bartha produces gH (UL22) comparable to that in virulent strains. PRV Becker displays slightly higher and/or differentially glycosylated levels of gH than the other two strains. Levels of the capsid protein VP5 (UL19) are shown for comparison and as a loading control. B) Ratio of gH vs. VP5 in each sample, using the ImageJ Gel Analyzer module. Equivalent amounts of protein were loaded in each lane. The blot was cut, with VP5 measured on the upper half and gH on the lower half to demonstrate equivalent levels of infection in each lysate. The same lysates were used for the analyses in Figure S3 (in Text S1); these are representative of three separate experiments. The conditions required to visualize the two bands of gH precluded measurement of cellular actin on the same blot. Positions of a standard marker are noted on the left.

Several unique Bartha mutations are located within functional domains of proteins not previously considered to affect Bartha's virulence and spread phenotypes, including gH (UL22), gB (UL27), and gN (UL40.5). The core fusion process of most alphaherpesviruses consists of receptor binding via gD (US6), followed by fusion mediated by gB (UL27) and the gH-gL (UL1) heterodimer. PRV gH has recently been crystallized, as have the homologous gH proteins of HSV-2 and Epstein-Barr virus (EBV) [68]-[70]. PRV Bartha has a Pro438Ser change in gH. In the recent crystal structure of PRV gH, this proline was highlighted as a key residue, because it mediates a bend at the end of an alpha helix in the gH core (domain III), which is necessary to allow one of four disulfide bonds in the protein [70]. This proline and the neighboring disulfide-bonded cysteine are absolutely conserved across all known herpesvirus sequences, including the evolutionarily distant beta- and gamma -herpesviruses [70]. In Western blot analysis of infected cell lysates (Figure 4), PRV Bartha produces two bands of gH protein that are comparable to those of the PRV Kaplan and Becker strains. There is no obvious difference in gH produced by these PRV strains.

We also detected three changes to the key fusion protein gB (UL27) coding sequence in PRV Bartha, which affect several residues immediately adjacent to gB's furin cleavage site (Ser506Ala, Pro507Ala, and Pro509Gln). Furin cleavage of gB has been shown to affect cell-cell spread of PRV and *in vivo* virulence of VZV [71], [72]. Transfer of just 11 AAs surrounding this furin cleavage site, corresponding to residues 497–507 of the PRV Kaplan gB sequence (PAAARRARRSP), are sufficient to confer protease-cleavage when inserted into PRV gC [73]. As noted previously [31], gB is still cleaved in PRV Bartha-infected cells *in vitro* (Figure S3 in Text S1), but it is unknown whether these changes in gB affect cleavage efficiency or other aspects of gB function in specialized cell types such as neurons.

Finally, PRV Bartha has a Leu7Pro alteration in the signal sequence of gN (UL49.5) that may affect glycoprotein processing and/or packaging [62], [74]. A previously detected Leu14Pro difference in Bartha's gC also affects the signal sequence, leading to inefficient maturation of gC, and reduced incorporation of gC into virions [62]. PRV gN is normally packaged into virions and affects the rate of virion penetration into cells [74], [75]. If this signal sequence mutation affects gN maturation or virion inclusion in a parallel way to that of the gC signal sequence mutation, it may well contribute to the delayed penetration kinetics and cell-to-cell spread phenotype of the attenuated PRV Bartha vaccine strain.

### Amino acid variation between strains of PRV, HSV-1, and VZV

**Figure 5:**
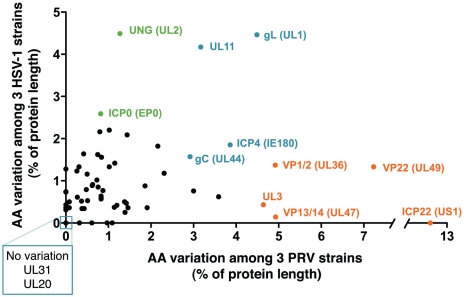
Inter-strain diversity in protein coding sequences, in PRV as compared to HSV-1. The total number of amino acid differences between three strains of PRV (strains Kaplan, Becker, Bartha) is normalized for protein length and plotted with data for the homologous proteins of HSV-1 (strains 17, F, H129) [81]. Blue color highlights variable proteins where inter-strain variation reaches similar levels in PRV and HSV-1, while orange highlights proteins that are much more variable in PRV than HSV-1, and green highlights the converse. Boxed proteins UL31 and UL20 show no variations in these six strains of alphaherpesvirus; UL31 also shows no coding variation across 18 strains of VZV [78], [79]. Proteins without homologues in both viruses are excluded, as are proteins in the Bartha deletion region. Table S6 lists all protein names, lengths, and percent variation in PRV, HSV-1, and VZV strains.

The genomes of alphaherpesviruses have long been thought to be quite stable with limited sequence variation among strains [76], [77]. This idea was well supported when the genome-wide comparison of 18 VZV strains revealed inter-strain coding variation of 1% or less [78], [79]. The four HSV-1 genome sequences available show modestly increased inter-strain protein-coding variation [80]-[83]. Surprisingly, we find that protein coding variation between PRV strains is higher than that observed for either HSV-1 or VZV (average of 1.6% for PRV, vs. 1.3% for HSV-1 or 0.2% for VZV; Figure 5 and Table S6) [78], [81]. When the coding sequences for each protein of these three new PRV genomes are compared, the inter-strain variation in AA sequence (number of AA residues varying between strains, normalized for protein length) reaches as high as 13%. Starting on the low end of variation, we found eight invariant proteins across these PRV strains (Figure 3), including the viral DNA polymerase UL30, the minor capsid proteins VP19c (UL38) and VP23 (UL18), the nuclear egress components UL20, UL31, and UL37, and the functionally uncharacterized proteins UL24 and UL56 (ORF-1). In contrast, ICP22 (US1) displays 13% inter-strain variation; this protein has transactivating and regulatory functions in related alphaherpesviruses [84], [85], but has only been studied at the level of transcript expression in PRV [86], [87]. In a similar comparison of AA sequence differences between 3 strains of HSV-1, the inter-strain variation peaked at 7% (for ICP34.5 (RL1) and US11; Table S6) [81]. VZV strains show even less variation in protein coding sequences, with a maximum of 1.2% AA variation (in ORF-1) between strains, and just two additional proteins with variation greater than 0.5% [78]. One of these two VZV proteins is ORF 62/71, which is homologous to PRV IE180 and HSV-1 ICP4; this protein is among the most variable across all known strains of these alphaherpesviruses. IE180 is the sole gene expressed with immediate-early kinetics in PRV, and is a key transactivator of viral gene expression [88]–[90]. In contrast, the nuclear egress proteins UL20 and UL31 thus far shows no inter-strain variation in all known genomes of PRV and HSV-1, while UL31 shows zero coding variation in VZV as well.

A comparison of the inter-strain variation in homologous proteins of PRV and HSV-1 (Figure 5 and Table S6) highlights several proteins that appear to vary more substantially in one virus than the other. Although ICP22 is the most variable protein in PRV, it is completely invariant among HSV-1 strains 17, F, and H129, as well among the previously described 18 strains of VZV [78], [79], [81]. Likewise, the viral egress protein VP13/14 (UL47) is among the most variant in PRV, but it is well-conserved in HSV-1, while the opposite is true for HSV-1 proteins uracil-DNA glycosylase UNG (UL2) and the ubiquitin E3 ligase ICP0 (EP0) (Figure 5, orange vs. green highlighting). Several proteins, which do not have homologs between HSV-1 and PRV, are also highly variable; these include PRV's viral egress protein UL3.5, which has the third-highest variability of PRV proteins after ICP22 and the tegument protein VP22 (UL49), and the two most variable HSV-specific proteins, which are the neurovirulence-associated protein ICP34.5 (RL1) and the PKR-antagonist US11.

### Short sequence repeats (SSRs) are prevalent in the PRV genome

**Figure 6:**
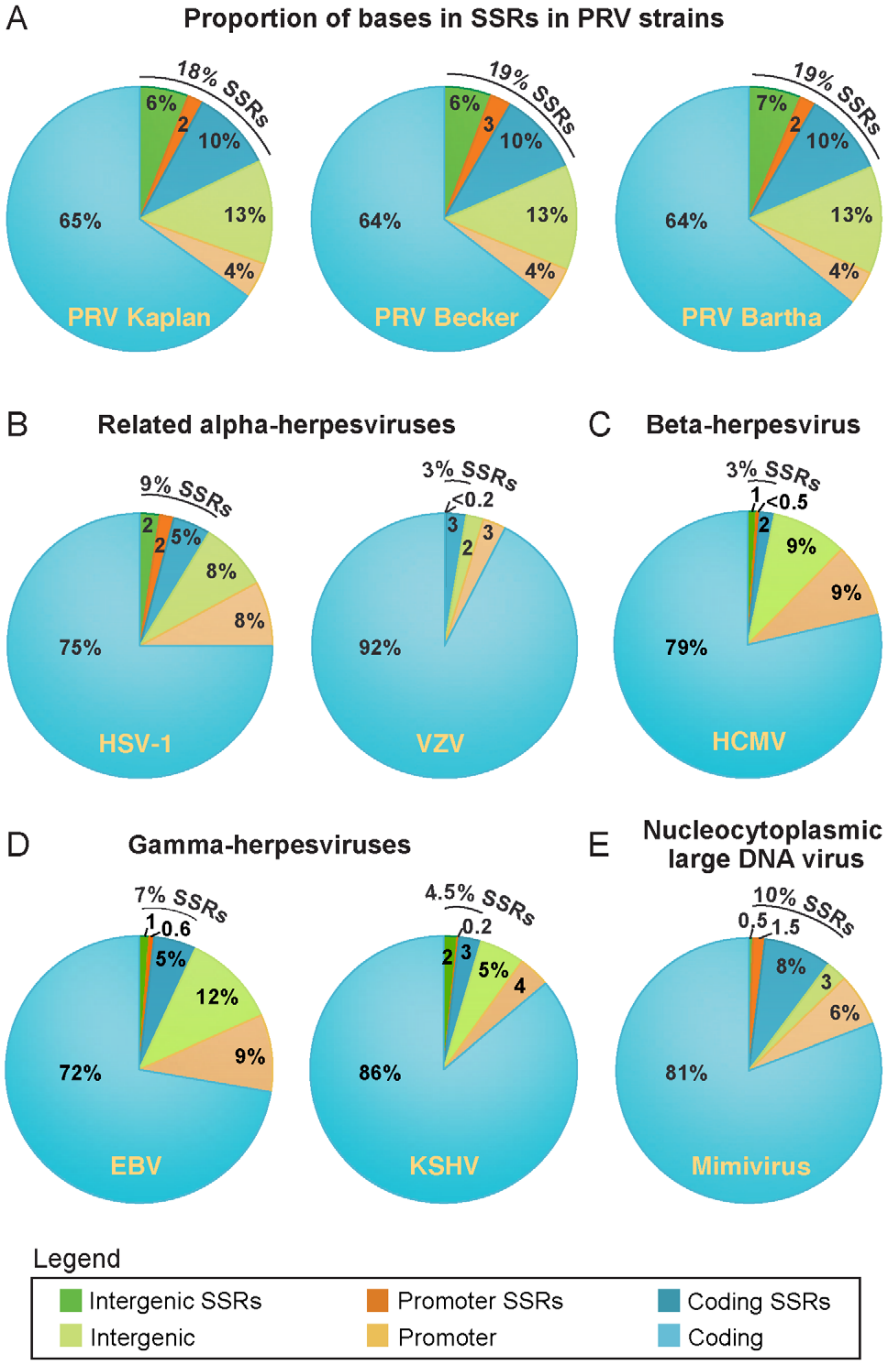
Prevalence of SSRs in PRV strains and in related DNA viruses. The proportion of bases in each genome involved in SSRs was calculated for (A) all three PRV strains, as well as for (B) the related human alphaherpesviruses HSV-1 and VZV, (C) the betaherpesvirus HCMV, (D) the gammaherpesviruses EBV and KSHV, and (E) a nucleocytoplasmic large DNA virus, Mimivirus. Pie charts depict what proportion of each genome falls into coding regions, promoter regions (defined as 500 bp upstream of a coding sequence), or open intergenic regions. Exact numbers and types of SSRs per genome are found in Table 4. A complete list of all PRV SSRs is found in Table S7.

SSRs are widespread in eukaryotic genomes, and mediate functional effects by serving as DNA-binding domains in promoters, protein folding motifs in coding sequences, and sites of inter-molecular recombination [44]–[47]. Since AA repeats generated several examples of inter-strain coding diversity above (Tables 1–3), we investigated the prevalence of SSRs in the PRV genome. SSRs are generally grouped into three main categories: homopolymers, which include a short run of the same base; microsatellites, where the repeating unit is less than 10 bases; and minisatellites, which have a repeating unit of 10–500 bases [44], [45]. The initial description of the PRV genome mapped 26 minisatellite SSRs using a DNA identity scoring matrix [30]. Using software designed to identify all size classes of SSRs and include both perfect and imperfect repeats (see Methods for details), we detected a significantly larger number of repeats, a total of 953 distributed across the PRV Kaplan genome (Table 4 and Table S7; minimum homopolymer length 6). SSRs in PRV occur in both coding and non-coding regions, promoters and open intergenic space, with similar proportions in all three PRV strains (Table 5 and Figure 6A). SSRs of all size classes are distributed throughout the genome, with a slightly higher accumulation of all types in the IR-US-TR region (Figure 1C and Figure S4 in Text S1). The majority of all SSRs in PRV (62%) contain triplet-based repeats (*e.g.* the repeat unit is a 3-mer, 9-mer, 21-mer, etc.). Likewise, 69% of homopolymers have a triplet-based length. Half of all SSRs are in coding sequences (474/953), and these are largely triplet-based (72%). Triplet-based repeats, as well as insertions or deletions (indels) and partial repeat units of non-triplet-SSRs, help preserve the coding content in the SSR-laden PRV genome because variation in these repeats (addition or removal of repeat units) does not change the reading frame of the downstream sequence.

**Table 4:** Comparison of ORF and SSR quantities in PRV, HSV-1, VZV, and Mimivirus.

Virus (official acronym) a	Strain b	Length (bp)	% G/C overall (in SSRs)	# proteins c	# mini-satellite SSRs (≥10 bp)	# micro-satellite SSRs (<10 bp)	# homo-polymers	Ref.
PRV (SuHV-1)	Kaplan	140,377	74% (79%)	69	382	230	341	d
HSV-1 (HHV-1)	17	152,261	68% (84%)	77	142	103	661	^e^
VZV (HHV-3)	Dumas	124,884	46% (47%)	73	15	17	282	^f^
HCMV (HHV-5)	Merlin	235,646	57% (57%)	165	104	49	343	^g^
EBV (HHV-4)	Raji	171,823	59% (77%)	94	71	23	423	^h^
KSHV (HHV-8)	GK18	137,969	54% (62%)	86	32	13	225	^i^
Mimivirus (APMV)	Mimivirus	1,181,549	28% (20%)	979	1,220	230	5,265	^j^

a Suid herpesvirus (SuHV), Human herpesvirus (HHV), and Acanthamoeba polyphaga mimivirus (APMV).

b NCBI nucleotide records: PRV Kaplan, JF797218; HSV-1 17, NC_001806; VZV Dumas, NC_001348; HCMV Merlin, NC_006273; EBV Raji, NC_007605; KSHV GK18, NC_009333; Mimivirus, NC_014649.

c Genes with duplicate copies in IR/TR are counted at both sites of occurrence. Reference sequences and protein data are from the NCBI Viral Genomes Project [185].

d This work,^ e^ HSV-1 [82], [83], ^f^ VZV [186], ^g^ HCMV [187], ^h^ EBV [188]–[192], ^i^ KSHV [193], ^j^ Mimivirus [125].

**Table 5:** Comparison of selected short sequence repeats (SSRs) in PRV strains Kaplan, Becker, and Bartha.

ID_strain, genome position_ a	Location^b^	Kaplan# units	Becker # units	Bartha# units	Repeat unit consensus (unit length) c
**Intergenic:**					
SSR_Ka151_	**left terminus**	3	NF d	NF	TACCTGGCACCCTGCCAACCCCAATCCCCCTCC (33mer)
SSR_Ka2093_	**between ORF-1 & UL54**	8.3 e	NF	6	G**GGGAG**ATG**GGGAG**AGGAGAT (21 mer)
SSR_Ka15795_	**between UL46 & UL27**	13.7 e	59 e	18.4 e	ACG**GAGGG**GA**GAGGG** (15 mer)
SSR_Ka31884_	**between UL35 & UL36**	5.6	2.7	4.8	CCCCAA**GTCCC**CCAATCC (18 mer)
SSR_Ka62103_	**between UL22 & OriL**	6.3	NF	NF	CG**CCCTC**T**CTCCC**AC (15 mer)
SSR_Ka62261_	**between UL22 & OriL**	6.5	10.5	11.5 e	AAG**GGGTC**TCT (11 mer)
SSR_Ka79207_	**between UL11 & UL10**	6.4	7.4	8.1 e	TGG**GGGAG**AGGA (12 mer)
SSR_Ka94997_	**between UL1 & EP0**	17	18	15	GGAGCA (6 mer)
SSR_Be100922_	**between left edge IR & IE180**	2.1	3	3	CCCCCCCCCCCATTTGCATATGACCGCTTCCCCCGGACGTGACGCTCGGG (50 mer)
SSR_Be101633_	between left edge IR & IE180	3.3	3.1	NF	GACCACCGGGACCACCAACACCGTCTACCTCCCACCAG (38 mer)
SSR_Ka106596_	promoter f: IE180	3.2	NF	3.2	CGGCCAATGGGATTTCTCTCGCCAACTTCCTCTCGCGTCTACTTTGCATGTCCGGCCCCCGCGGCGGCCATCTTGGCCCCTCGA (84 mer)
SSR_Ka107138_	**between IE180 & OriS (in IR)**	12.5 e	4.1	8.8	TGT**GGTGG**TCTCTGTGTTG (19 mer)
SSR_Ka115377_	**between US1 & edge of IR**	3.1	NF	NF	G**GGGAG**TGGGATGGGG**GTGG**AGAC**GGTGG**A**GGGAG**A (36 mer)
SSR_Be115911_	**between US1 & edge of IR**	NF	20.6 e	NF	GGTG**GAGGG**A**GAGGG**GGAC (19 mer)
SSR_Ka115550_	**promoter: US3**	9	3	10	GG**GGGAG**TCC (10 mer)
**In coding sequences:**					
SSR_Be33478_	UL36	1.4	5.2	5.4	GGGGCCGGCCGCGAAGGTGGT (21 mer)
SSR_Ba32980_	UL36	1.1	NF	3.1	GGCCGGCCGCGAAGGTGGTGGGGCCGGCGGTGGTGC (36 mer)
SSR_Ka57529_	UL25	3.2	3.2	3.2	CCTCGGGCGCCTCCTCGGCGGCGCGCG (27 mer)
SSR_Ka114728_	US1	72.8	63.8	80.2	CGAGGA (6 mer)

a Repeats selected have a TRF alignment score ≥100 and/or VarScore ≥1, with a repeat unit length ≥6 and ≥3 repeat units. The PRV Kaplan genome was primarily used for repeat screening, with additional searches run on the other genomes to detect SSRs with high scores in Becker or Bartha but not Kaplan. SSR identifier (IDs) denote the strain name where the SSR was first detected (Ka, Kaplan; Be, Becker; or Ba, Bartha) and the start position on that genome. For clarity, only the IR copy of SSRs falling into the large IR/TR repeats is shown (see Table S7 for a full listing of all SSRs).

b Boldface indicates SSRs previously noted in the initial description of the mosaic PRV genome [30].

c Boldface indicates CTCF binding sites within these SSRs, as defined by Amelio *et al.* [104].

d NF, not found. Indicates that a homologous repeat was not found in this strain or had diverged beyond detection.

e CAPRE was used to estimate repeat unit length of these perfect SSRs. See Figure S1 (in Text S1) and Methods for details.

f Promoter refers to sequences within 500 bp upstream of a start codon.

All coding sequences, except the small UL11 gene, contain SSRs (Figure 1C). However it is interesting to note that nineteen genes are free of homopolymers, a size class where expansion or contraction of the SSR is likely to disrupt the reading frame (Table S7). Likewise another 20 genes have regions of at least 1 kb that are homopolymer-free. For instance, the large tegument protein VP1/2 (UL36; 9.2 kb in length) has no homopolymers in its initial 5.5 kb (Figure 1A,C), which contains several domains affecting capsid transport, replication, and neuroinvasion [91]–[95]. In contrast, VP1/2's homopolymer-rich C-terminal region has been previously shown to be dispensable for viral replication [96]. Of the 25 core genes found across multiple families of *Herpesviridae* that are essential for growth in cell culture [76], 18 have no homopolymers or regions >1 kb that are homopolymer-free. As additional sequences become available for phylogenetic comparison, it may be possible to determine whether this is a chance occurrence or the result of purifying selection.

Since SSRs have not been comprehensively examined in other DNA virus families, we extended these analyses to include the genomes of a wide variety of human herpesviruses, including HSV-1, VZV, HCMV, EBV, and KSHV (Table 4 and Figure 6B–D). To ascertain if these results hold for non-nuclear, non-mammalian viruses, we selected as an outgroup for comparison the nucleocytoplasmic large DNA virus Mimivirus, which infects pathogenic amoebae (Figure 6E) [97]. PRV has the highest overall SSR burden, with short repeats encompassing 18% of the genome, which is roughly double the proportion found in HSV-1, EBV, and Mimivirus, and 5–6 times that of VZV, HCMV or KSHV. In all of these viruses, more than half the SSRs fall into coding regions (Figure 6), creating potential effects on protein structure if these SSRs vary in length between strains. SSRs also occupy a noticeable fraction of the intergenic and promoter regions in PRV and other genomes (Figure 6). For those genomes with a biased nucleotide content, the bias is exaggerated in SSRs (Table 4). PRV's overall genome is 74% G/C, but this level is 79% when all SSR sequences are pooled together. This is similar in HSV-1 (68% G/C overall; 84% in SSRs) and EBV (59% G/C overall; 77% in SSRs), and mirrored in reverse in the A/T-rich genome of Mimivirus (72% A/T overall; 80% in SSRs). PRV thus provides a rich set of SSRs for analysis of a phenomenon that extends to many other viruses.

Previous work in yeast, humans, and other organisms has demonstrated that variation in SSR length, either between individuals or during evolutionary adaptation, can result in phenotypic effects [47]–[50]. Although the overall proportions of SSRs are similar in the PRV Kaplan, Becker, and Bartha genomes (Figure 6A), a comparison across PRV strains revealed that homologous SSRs vary in length between strains (Table 5). Previously, variation in a selection of microsatellites (≤6 bases in length) has been shown for HSV-1, HCMV, and HIV [98]–[100], but the genome-wide complement of all SSR types has not been analyzed. The comparison of homologous SSRs reveals that not all SSRs can be recognized in all three strains (*e.g.* SSR_Ka151_, SSR_Ka2093_, and SSR_Ka62103_ in Table 5). However the majority of those that do occur in all strains vary in the number of repeating units (of 861 SSRs found in all three strains, 539 vary in number of repeating units). If these SSRs contain transcription factor binding sites or occur in protein coding regions, then these inter-strain differences in SSR copy number may influence gene expression or protein folding domains, and thus lead to phenotypic differences between strains.

### Inter-strain variation in SSRs containing CTCF DNA-binding sites

One of the best characterized biological roles for SSRs in herpesviruses are the CCCTC-binding factor (CTCF) binding sites that flank latency-associated transcripts in the genomes of HSV-1 and the gammaherpesviruses EBV and KSHV [101]–[108]. In each of these cases, CTCF binds to motifs within SSRs found near loci that are transcriptionally active during latency; this interaction is proposed to have chromatin insulating and/or silencing effects that maintain a repressed state in flanking genes. CTCF-binding sites occur in several additional conserved locations throughout alphaherpesvirus genomes, as shown by Amelio *et al.* in a comparison that included HSV-1, VZV, and PRV [104]. Because many PRV SSRs showed inter-strain variation in copy number or length, we investigated CTCF-binding sites in PRV Kaplan, Becker, and Bartha. Of the 17 CTCF binding sites mapped by Amelio *et al*, 12 were mapped as falling into SSRs in our inter-strain comparison (Table 5; CTCF-binding sites in the repeat-unit consensus are underlined and in bold). All of these vary in repeat-unit length between strains (*e.g.* Table 5: SSR_Ka31884_, SSR_Ka115550_). Although several have diverged enough to be listed as separate SSRs, their overall location and CTCF-binding ability are preserved (*e.g.* Table 5: SSR_Ka115377_ and SSR_Be115911_; see Table S7 for orthologous SSR_Ba115943_). The greatest inter-strain variation in SSR length occurs at SSR_Ka15795_, between UL46 and gB (UL27), where PRV Becker has three times as many repeating-units as either PRV Kaplan or Bartha. This SSR contains both CTCF-binding sites and a non-canonical Egr1/2 binding site, both of which have repressive effects on expression of nearby genes in HSV-1 [57], [104], [109]–[111]. Initial studies show that gB levels in PRV Becker-infected lysates do not appear significantly lower than those in PRV Kaplan or Bartha (Figure S3 in Text S1). Further work will be required to determine if the flanking SSR length affects gB expression and function.

In the only previous publication comparing full-length genomes of HSV-1 (strains 17, H129, and F), the length of fourteen major SSRs throughout the genome were not determined and were instead set to match the reference genome length [81]. These fourteen SSRs, classically termed reiterations in the HSV literature [37], [38], [82], [83], correspond to the fourteen CTCCC-domain-containing SSRs defined by Amelio *et al.* [104]. To discern if inter-strain variation such as that observed in the PRV genomes is found in HSV-1 as well, we PCR amplified and sequenced two of these SSRs from the HSV-1 strains F and H129. Both SSRs displayed inter-strain variation in copy number, with the reference strain 17 (GenBank Accession NC_001806) having more SSR units at both sites than either the clinical isolate H129 or the laboratory strain F (IRS reiteration 3 [CTRS3 in Amelio *et al.*]: 6.5 copies in strain 17, 4.7 copies in H129, 1.7 copies in F; US reiteration 1 [CTUS1 in Amelio *et al.*]: 10 copies in strain 17, 2 copies in H129, 2 copies in F). These data suggest that inter-strain variation in SSR length may affect CTCF-binding efficiency in HSV-1 and could contribute to inter-strain differences in related phenotypes.

### Estimation of selected SSRs by Coverage Adjusted Perfect Repeat Expansion (CAPRE)

Annotation of SSRs in the draft PRV genome assemblies had revealed several discrete areas in each genome where peaks of very high coverage coincided exactly with perfect SSRs: for example a peak of over 100,000-fold coverage around an SSR at position 15,600 in the PRV Becker genome (Figure S1 in Text S1 and Table 5). This very high coverage (>2 standard deviations above the median) occurred at three SSR sites in PRV Kaplan, three SSRs in PRV Becker, and four SSRs in PRV Bartha. (Figure S1 and Table S3 in Text S1, also noted in Table 5). *De novo* assembly methods cannot distinguish whether repeated sequence reads originate from perfect, extended copies of an SSR unit, or from additional coverage depth of a single unit, and the software therefore creates a final assembly with the minimal number of repeating units supported by the data [112]. In fact, the high coverage peak in PRV Becker coincides with the largest SSR array of perfect repeats in the original mosaic PRV genome, which had 39 copies of a 15-mer at this site [30], suggesting that this peak might result from *de novo*-assembly compression of the homologous SSR in PRV Becker. The short unit size of this SSR (15-mer) meant that its copy number could only be estimated by RFLP and Southern blotting, and the likely amount of perfect repeating units could lead to laddering and polymerase slippage errors in PCR analysis. We therefore devised an approach to computationally estimate the length of these perfect tandem repeats that demonstrate potential compaction during assembly, in order to facilitate future HTS-genome assemblies and preserve coverage-based information on inter-strain variation in SSR length.

Coverage-Adjusted Perfect Repeat Expansion (CAPRE) is based on methods used for copy number variant estimation in HTS data [113], [114], which is used in larger genomes to detect duplications of chromosome regions or individual genes. As in copy number estimations, CAPRE takes into account the observed coverage depth and estimates the length of intergenic SSRs based on the expected sequence depth for its G/C nucleotide content (Figure S2A in Text S1). In order to estimate SSR length conservatively, CAPRE predicts SSR length based on the median coverage expected for a given G/C content, and can also be used to predict potential upper- and lower-range estimates based on the upper and lower quartile ranges of this coverage (Figure S2A in Text S1). Because it is imprecise, we applied this method sparingly, and used it only at intergenic sites where coverage depth exceeded two standard deviations from the median and coincided with a perfect SSR. We used CAPRE to expand the lengths of three SSRs in PRV Becker, three in Kaplan, and four in Bartha (Figure S1 and Table S3 in Text S1). This did not affect the overall count of SSRs in Table 4, but did affect the length of several SSRs included in Table 5 (*e.g.* SSR_Ka15795_; these are marked). We incorporated these CAPRE-expanded SSRs into the overall assembly of each genome before final annotation and comparisons. The CAPRE method provided a means to estimate the length of these repeats and yielded a more even distribution of sequence read coverage at these sites in the final genome (Figure S1 in Text S1).

To test whether the CAPRE script provides a reasonable estimation of SSR length, we compared the CAPRE-expanded SSRs to alternative sources of data on actual SSR length. First, we compared the three CAPRE-expanded SSRs of PRV Kaplan (Table S3 in Text S1) to their counterparts in the original PRV mosaic genomes. Each of these SSRs falls into areas of the mosaic genome that were originally derived from the Kaplan strain, facilitating comparison of our estimated lengths to SSR lengths that were determined in strain Kaplan by traditional Sanger sequencing. For SSR_Ka107138_, the CAPRE-estimated length nearly matches that of the Sanger-sequenced Kaplan isolate (12.5 copies here vs. 10.5 copies in the mosaic), while for the other two it provides a conservative under-estimate (SSR_Ka2093_ is 8.3 copies here but was 17.3 in the mosaic; SSR_Ka17595_ is 13.7 copies here, but was 39 copies in the mosaic).

**Figure 7:**
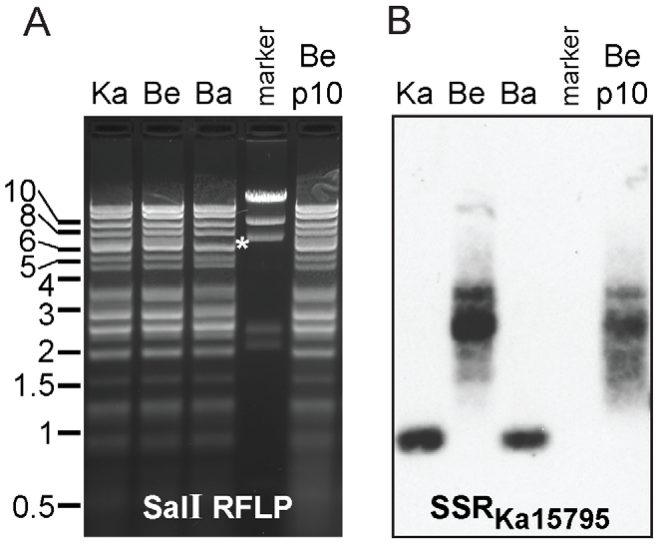
Southern blot of CAPRE-estimated SSR lengths. A) RFLP analysis of SalI fragments of PRV strains Kaplan (Ka), Becker (Be), Bartha (Ba), and a Becker stock passaged 10 times *in vitro* (Be p10). Positions of one standard marker are noted on the left; another marker lane is shown between the Bartha and Becker p10 lanes (5 bands: 23 kb, 9.4 kb, 6.6 kb, 2.3 kb, 2 kb). Asterisk (*) at 7 kb in the PRV Bartha lane highlights a size shift in the fragment containing the Bartha US-region deletion. B) Southern blot of the same fragments, using a biotinylated probe matching SSR_Ka17595_ (a perfect 15-mer) to reveal the size of the SalI fragment containing this site. Without any SSR content, this fragment would be ∼0.55 kb in PRV Kaplan and Bartha, and ∼0.74 kb in PRV Becker. Based on observed fragment sizes, the 1 kb Kaplan and Bartha fragments each have ∼30 copies of this SSR, while the 2.5 kb average Becker fragment (range 1.6–3.5 kb) has on average ∼120 copies (range 58–184 copies). The variable fragment size in PRV Becker shifts upon passage *in vitro* (Be p10).

Next, we used RFLP and Southern blot analysis to estimate the length of the most divergent SSR between strains (Table 5, SSR_Ka15795_); this SSR is also the only one expanded by CAPRE for all three strains (Table S3 in Text S1; SSR_Ka15795_, SSR_Be15739_, SSR_Ba15751_). We hybridized a probe to this SSR against SalI-digested DNA from PRV Kaplan, Becker, and Bartha (Figure 7). The size of the SalI fragment reflects a much larger size in PRV Becker than in Kaplan and Bartha, and further reveals that this SSR varies in length even within the purified PRV-Becker stock. A prior Southern blot analysis by Simon *et al.* showed that this same SSR varied in length between strains and within plaque isolates of a given PRV strain [115]. As occurs here with strain Becker, those authors found that the strain Phylaxia had a wide and blurry band of probe hybridization, while other PRV strains (Kaplan and Dessau) had tight bands [115], suggesting strain-specific differences in SSR length stability. To investigate the stability of this SSR, we serially passaged the plaque-purified PRV Becker stock ten times in culture (potentially 20–30 cycles of replication at low multiplicity of infection (MOI); see Methods for details). RFLP analysis of this stock, termed Becker p10, differed from the parental PRV Becker only in the classically variable BamHI fragments 10 and 12 (Figure 2B and 7A), which have been shown to vary with repeated passages [22], [35], [41], [42]. However the band distribution of SSR_Be15739_ shifted slightly in the Becker p10 stock (Figure 7). The upper length estimate for SSR_Be15739_ (Table S3 in Text S1) falls into the band distribution observed in Figure 7B, and the predicted ratios across strains (Table 5) likewise mirror the observed differences. Thus the CAPRE script met our goal of conservative length estimation, and allowed correct prediction of the extreme inter-strain size differential of the homologous SSR that falls between UL46 and gB (UL27).

### PCR validations reveal homopolymers as mutational hotspots

We also used PCR sequencing to refine and validate selected areas in the assembly (Tables S2 and S4, and Figure S1 in Text S1). The majority of these PCR products confirmed divergence in the newly sequenced strains from the previous mosaic reference genome, while the remainder corrected SSR-based issues in the assembly, *e.g.* for Becker UL3.5 and VP1/2 (UL36), and Bartha VP1/2 (Tables 1–3 and Table S2 in Text S1). To assess sequence stability in PRV genomes over time, we PCR-amplified and sequenced the same regions of parental stocks of these plaque-purified isolates. We found no base pair differences between 8.8 kb of the parental and progeny genomes, in ten spatially distributed PCR comparisons (Table S2 in Text S1).

We and others have previously demonstrated that direct Sanger sequencing of PCR products, vs. cloning and subsequent sequencing, provides useful and sensitive detection of minority variants in a population [78], [81]. In a prior sequencing study, we detected variation at a C_6_ homopolymer in an HSV-1 stock; plaques picked from this stock reproduced either homogeneous C_6_ or C_5_ variants [81]. Although we were not searching for minority variants, all of the above PCR sequences were visually screened for any evidence of such variation. We detected two such sites, one each in PRV Becker and Bartha, in different homopolymers upstream of ICP22 (US1). ICP22 has a high concentration of homopolymers in its upstream region (Figure 1A,C). At a C_10_ site upstream of ICP22, the majority of the PRV Becker PCR products reflected a homopolymer length of ten, while a minority of the products had a length of nine (Figure S2C in Text S1); these may represent the contributions of viral nucleocapsid DNA population used as a template. Likewise, at a different C_10_ homopolymer upstream of ICP22, PCR sequencing of PRV Bartha revealed homopolymer variants of nine, ten, and eleven (data not shown). Although these variants could reflect polymerase slippage during PCR or Sanger-sequencing of the PCR products, both PCR products contain nearby C_8_ homopolymers that show no minority products. The homopolymer variants described here, along with accumulating evidence from other alphaherpesviruses, suggests that homopolymers are mutational hotspots in PRV as well [78], [81], [116]–[119].

### Sequence polymorphisms in plaque-purified and passaged strains

**Figure 8:**
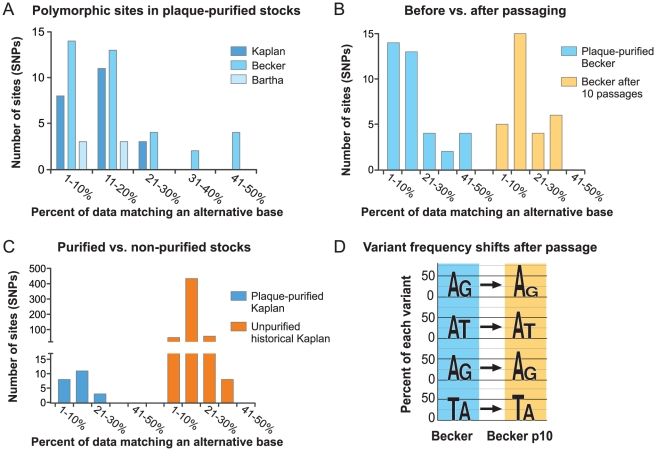
Percent of data supporting polymorphic base calls in PRV genomes. A) A limited number of polymorphic bases were detected in the plaque-purified strains Kaplan, Becker, and Bartha; these were analyzed to deduce the percent of sequence data supporting the primary vs. alternative base calls. The majority of polymorphic sites show 1–20% support for the alternative base call, or 99–80% support for the primary base call. B) After passaging the purified Becker stock multiple times *in vitro* (Becker p10), there was no increase in the overall number of polymorphic bases, and only a slight shift in the degree of support for alternative base calls. C) However in an unpurified historical stock of PRV Kaplan, which is the parent of the plaque-purified stock used for sequencing, hundreds of polymorphic bases were observed. Despite the larger quantity of polymorphic bases, the degree of support for the alternative base is similar to that found in the plaque-purified strains. D) Graph displays the alternative base calls for the four most variant polymorphisms in PRV Becker (41–50% bin), and how these specific bases were called in the Becker p10 progeny stock (the height of each base letter corresponds to its frequency). These sites display shifts in base frequency in the Becker p10 stock.

There is limited evidence for sequence polymorphisms in large DNA virus genomes; these include several studies that noted SSR-based variation in clonal stocks of herpesviruses [35], [78], [120], several recent studies of variation in HCMV DNA from both clinical and lab-passaged strains [121]–[124], and the recent observation of a small number of polymorphic bases scattered throughout the large DNA genome of Mimivirus [125]. We therefore used single-nucleotide polymorphism (SNP) detection software to check for any variation in base calls when HTS data from each strain were aligned back to the finished genome (see Methods for details). A small number of bases (0.004–0.03% of each genome) were indeed called as polymorphic in each plaque-purified isolate (22 in PRV Kaplan, 37 in Becker, 6 in Bartha). Unlike HTS genomes with low coverage depth, HTS data for these viral genome sequences provides deep coverage and a strong likelihood that these base variations are not sequence errors. An examination of the percent of reads contributing to each polymorphic base calls revealed that in most cases, the alternative base was present in a minority of the sequence reads, from 1–20% (Figure 8A).

PRV Becker was the only strain with several polymorphic bases approaching 50–50 variation in the primary versus the alternative base (Figure 8A). We therefore investigated the stability of these polymorphic bases in the serially passaged Becker p10 strain. Nucleocapsid DNA from the Becker p10 stock was sequenced and aligned to the PRV Becker genome for SNP analysis (see Table S1 in Text S1 for details of HTS data generated). We found no increase in the overall number of polymorphic base calls after serial passage (Becker: 37, Becker p10: 30), and only a slight shift in the frequency of observation of the secondary base call (Figure 8B). Many polymorphic sites in the Becker p10 stock (28 of 30) were in the same position as in the parental, purified Becker stock but had shifted in allele frequency. An additional 9 polymorphic sites either were lost or gained during the passaging that produced the Becker p10 stock. The four most polymorphic sites in the original PRV Becker stock were still called as polymorphic in Becker p10, but had shifted in allele frequency (Figure 8D). Interestingly, only one SNP in any of these strains affected a coding sequence, and this one (P2172A) occurred in the proline-alanine rich region of Kaplan VP1/2 (UL36) that is dispensable for viral replication *in vitro* [95], [96]. The SNPs in these plaque-purified and limited-passage strains were almost exclusively located in non-coding regions.

Since serial passaging of a plaque-purified population had little effect on these polymorphisms, we examined variation in one of the non-purified viral stocks that gave rise to these plaque-purified isolates. Here we sequenced the oldest viral stock available in the lab, which is the parent of the plaque-purified PRV Kaplan used for these studies [62], [126]. RFLP profiles of this PRV Kaplan stock, termed Kaplan n.p. (not purified), matched that of the plaque-purified PRV Kaplan isolate (Figure 2B). HTS data for Kaplan n.p. was aligned to the PRV Kaplan genome and used for SNP calling (see Table S1 in Text S1 for details of HTS data generated). This stock possessed 547 polymorphic sites relative to the plaque-purified genome (0.39% of the genome; Figure 8C and Figure S5 in Text S1). As found for SNPs in the plaque-purified strains, most alternative base calls resulted from variants present at 1–20% (Figure 7C). Strikingly, the majority of these SNPs occur in coding regions, and are well-distributed across the PRV genome (Figure S5 in Text S1). Because these data cannot distinguish how many polymorphisms are present in any one viral genome of the Kaplan n.p. stock, versus distributed across the entire viral population in that stock, we cannot determine the extent of selection that occurred during plaque-purification. Future sequencing technologies that can examine single genomes will be required to address this. Together with the results above, we suggest that subtle variations such as these SNPs and homopolymer length variants provide the genetic diversity to help these strains adapt to future evolutionary pressures.

### Defining a new reference genome for PRV

The genome currently used as a reference for PRV is a mosaic of six strains [30] We therefore propose that the PRV Kaplan genome presented here (GenBank Accession JF797218) serve as a new reference genome for PRV. Strain Kaplan contributed 86% of the sequence in the mosaic reference genome, while the remainder included sequences from strains Becker, Rice, Indiana-Funkhauser, NIA-3, and TNL. Accordingly, we compared our complete PRV Kaplan genome to that of the original mosaic reference genome. Not surprisingly, the majority of protein coding differences between Kaplan and the mosaic genome (81%; 141 of 173 amino acid (AA) differences) occur in twelve of the thirteen proteins that were originally sequenced from non-Kaplan strains: gB (UL27), ICP18.5 (UL28), ICP8 (UL29), UL43, gC (UL44), TK (UL23), ICP0 (EP0), gG (US4), gI (US7), gE (US8), US9, US2 (see Table S5 in Text S1 for specific AA differences).

Several of these sequence differences significantly affect the resulting protein because of frameshifts in the strains used for the mosaic genome. The largest frame-shift changes 46 AAs in the extracellular domain of gG (US4), which has been mapped as a chemokine-binding region [67]. The gG sequence in the mosaic genome was derived from PRV strain Rice. Alignment of the three new PRV strain genomes, along with two geographically distinct gG sequences deposited in GenBank (Ea, China: AY319929, NIA-3, Ireland: EU518619), revealed that the PRV Rice strain included in the original mosaic genome is the only one to possess this frame-shift sequence and cannot be representative of most PRV strains. Similarly, all three new genomes share a common sequence of ICP8 (UL29; only 1 AA difference in PRV Becker; Table 3), which is a single-stranded DNA binding protein that functions in both replication and recombination of the viral genome [127], [128]. This new ICP8 sequence differs from the TNL strain sequence of ICP8 found in the mosaic PRV reference at a total of 20 residues (Table S5 in Text S1), including a compensated frame-shift that affects a stretch of 8 amino acids immediately flanking the zinc finger domain [129].

### Conclusions

#### Herpesvirus genomes: a microcosm of HTS eukaryotic genome assembly

Herpesviruses are among the largest DNA virus genomes and cause significant human disease, making the characterization of their sequence diversity a priority. While viral discovery screens using HTS often produce sufficient data to assemble entire RNA virus genomes [130]–[136], the ten-fold larger size of herpesvirus genomes means that only directed sequencing projects have thus far produced data on new strains [81], [120], [137], [138]. Herpesvirus genomes represent a microcosm of the features found in eukaryotic and bacterial genomes: abundant SSRs, histone modifications, splice sites, and microRNAs, among others, with frequent recombination at the large inverted repeats. An improved understanding of how these elements vary in these viral genomes may shed light on related sequence features in larger genomes, where sequencing of repeated generations or multiple related isolates may be prohibitive in cost or computational time. For instance, while the G/C coverage bias seen in these herpesvirus genomes has been previously observed in higher organisms [59], [60], there has not yet been sufficient depth of coverage and variety of G/C-rich sequence structures to correlate specific sequences with specific coverage-depth consequences. The G/C-bias of PRV and HSV-1 genomes along with their deep sequence coverage (>2,000-fold on average) provide data for future exploration of these issues, which will then provide insight relevant to all future sequencing endeavors.

#### Multiple glycoprotein mutations in the PRV Bartha vacfcine strain

Herpesvirus virions are coated in glycoproteins, which play a major role in viral spread from cell to cell and host to host, and are thus crucial to pathogenesis and vaccination strategies *in vivo* [8], [9], [139]–[141]. PRV has 11 glycoproteins, with functions including fusion (gH, gL, and gB), cellular attachment (gC, gD), rate of virion penetration (gN, gM), triggers of host immunity (gG, gI), viral transport in axons (gE), and virion egress (gK). The genome of PRV Bartha reveals mutations in genes that encode the majority of this suite of glycoproteins. Previously known changes in PRV Bartha that affect glycoproteins included the US-region deletion that removes gE and gI, a signal sequence mutation of gC, and a residue change affecting the N-glycosylation site of gM; all of these have also been shown to affect PRV Bartha's spread in culture, and the role of gE and gI have been confirmed to affect the attenuation of PRV Bartha's virulence *in vivo* [21], [28], [31], [61]–[65], [142]. To this list, we now add several mutations in the coding sequences of gN, gB, gH, gG, and gD, which are unique to PRV Bartha and are not seen in the virulent PRV Kaplan or Becker strains. Future work can now explore the relevance of these sequence differences to the attenuation of PRV Bartha's virulence *in vivo*, and their potential use in aiding the development of an HSV-1 vaccine strain.

#### SSRs and homopolymers fuel inter- and intra-strain diversity

Prior to this study, few PRV SSRs had ever been analyzed for potential inter-strain variation [86], [115], [143]–[145]. For decades, researchers have known that certain regions of the PRV and HSV-1 genomes are variable by RFLP analysis of repeatedly passaged virus stocks [22], [35], [42], [43], but little work has been done to elucidate the basis of this variation. The most variable sections of the PRV genome by RFLP analysis are located within BamHI fragments 10 and 12 (Figure 2), which represent the IR and TR copies of ICP22 (US1) and its upstream region. ICP22 has the highest inter-strain variability of any PRV protein (Figure 5). This region includes both areas of homopolymer length variation found in the plaque-purified strains (see above), has a large complement of SSRs of all size classes (Figure 1C), contains several SNPs in its flanking untranslated regions in every strain (Figure S5 in Text S1), and was highly refractory to PCR analysis (data not shown). Taken together, this region shows uniquely high variability that extends well beyond the prior RFLP observations. The ICP22 (US1) protein of PRV has been virtually unstudied at the protein level, so that further work is required to understand its role and the significance of its variability between strains [86], [87]. Our analysis thus reveals a likely target for the historical variability of restriction-digest fragments of this region of the PRV genome, and suggests that similar features could be associated with the classically-variable fragments of the HSV-1 genome as well.

Although larger SSRs are more noticeable to the eye, homopolymers of six or more consecutive bases are the most abundant class of SSRs in PRV and all viral genomes thus far examined. These numbers would only increase if we included homopolymers of five or fewer. Homopolymers have been previously suggested as mutational hotspots for HSV, but only in the context of two genes where they have been well-studied. First, resistance to the drug acyclovir and related nucleoside analogs is often mediated by changes in homopolymers of the TK (UL23) gene, an observation documented in several alphaherpesvirus species [117]–[119], [146]–[148]. Second, variation in the human antibody response to HSV occurs because of homopolymer mutations in the gG (US4) gene [149], [150].

We now suggest that homopolymers across the genome are mutational hotspots for evolutionary diversity in all alphaherpesvirus strains, and potentially in other virus families as well. Examples from the literature support this, with a wide array of examples mentioned in passing as part of other studies: the C_4_→C_6_ (wild-type→mutant) shift in HSV-1 strain 17 that caused early struggles in recognizing ICP34.5 (RL1) as a valid gene [151], a C_7_→C_6_ deletion in the *vhs* (UL41) gene of the HSV-2 HG52 strain [152], a T_7_→T_6_ mutation in UL5 of an attenuated Marek's disease virus genome [153], a spontaneous G_7_→G_8_ insertion in gE (US8) in an engineered strain of PRV [154], among others [78], [81], [99], [100], [155]–[158]. These examples, in conjunction with the clinical examples in TK and gG above, and our own data presented here, demonstrate the homopolymer mutations can occur throughout the herpesvirus genome. The aforementioned studies of TK and gG sequences in clinical samples demonstrate that homopolymer mutations occur readily during human infection. Together these data suggest that this highly abundant class of SSRs could provide a major source of adaptive variation for viral strain divergence. Beyond these viruses, homopolymer variation has been previously found in organisms from yeast to worms to humans [45], [159]–[162]. A significant proportion of cancer-associated mitochondrial DNA mutations occur at homopolymers [163]–[165]. As described earlier, changes in SSR length have been demonstrated to affect gene expression, protein interactions, and chromatin binding, among other functions [45], [47]–[50]. Future study of homopolymeric and SSR-based variation in herpesviruses may help to reveal the evolutionary fitness contributions of these mutational hotspots.

## Methods

### Virus stocks and passaging

PRV Bartha is a highly passaged vaccine strain, derived from the original Aujeszky strain which was isolated in Hungary [29]. PRV Becker is a virulent field isolate from dog, originally isolated at Iowa State University (USA), with subsequent laboratory passage [166]. PRV Kaplan is a virulent strain with extensive laboratory passage, likely derived from the Aujeszky strain [126], [167]. All viral stocks were grown and titered on monolayers of PK-15 pig kidney cells (ATCC cell line CCL-33). Stocks of each virus were triple-plaque-purified, expanded, and used to infect cells for a nucleocapsid DNA preparation. Viral nucleocapsid DNA was prepared by previously published methods [81], [168], [169].

A passaged PRV Becker strain (Becker p10) was produced by infecting a monolayer of cells with the plaque-purified stock at a multiplicity of infection (MOI) of 0.01. At full cytopathic effect (CPE), a small aliquot of this virus was used to directly infect a fresh monolayer of cells, and this procedure was repeated a total of ten times. The resulting stock was used to prepare nucleocapsid DNA for sequencing and RFLP analysis.

### Illumina library preparation and sequencing

DNA sequencing was carried out according to manufacturer protocols and reagents, using an Illumina Genome Analyzer II with SCS 2.3 software at the Princeton University's Lewis-Sigler Institute Microarray Facility. Five micrograms of nucleocapsid DNA was sequenced for each strain, using either one (PRV Kaplan, Becker p10) or two (Becker, Bartha, Kaplan n.p.) flowcell lanes. All sequencing runs were 75 cycles in length, except for one Becker and one Bartha lane of 51 cycles. The total number of sequence reads generated for each strain are listed in Table S1 (in Text S1). All Illumina sequence data has been deposited at the NCBI Short Read Archive under Accession ID SRA035246.1.

### Initial data processing and quality control

Initial data processing included several steps: 1) Illumina output converted to a standard file format, 2) library adaptor contaminants removed, 3) host genome sequences removed, 4) mononucleotide reads removed, 5) duplicate runs combined, and 6) quality and length trimming applied. All data and scripts described here are available at a genome-browser (http://viro-genome.princeton.edu) and data analysis website (http://genomics-pubs.princeton.edu/prv) hosted by Princeton University's Lewis Sigler Institute.

First, a script from the FASTX-toolkit developed by the Hannon lab (http://hannonlab.cshl.edu/fastx_toolkit/) was used to remove adaptor sequences resulting from the Illumina library preparation. Next, because these PRV viruses were grown in pig kidney cells, we used the Bowtie software package [170] to compare the sequence data against the *Sus scrofa* pig genome (NCBI build 1.1) and remove any sequences perfectly matching the host genome. The percent of contaminating host DNA is listed for each strain in Table S1 (in Text S1). Finally, we filtered out any reads that were entirely mononucleotides, which we previously found can confound genome assembly [81]. Finally, where relevant, we concatenated sequence data from two sequencing runs.

Two scripts were then used to remove poor-quality base calls from the end of the Illumina short-sequence reads. First, we used an adapted version of the quality-trimming script (TQSfastq.py) from the SSAKE *de novo* assembly software package [171]. We modified the parameters for quality threshold (T) and consecutive bases (C) above threshold, producing trimmed datasets for each strain with the default settings of T10, C20 or a more stringent quality control trimming of T20, C25. We then used the more stringently-filtered dataset as the input to a universal length trimmer from the FASTX toolkit, which truncated all sequences in the data file at a specified length, in this case either 41 or 51 bp. This generated four quality-filtered and trimmed datasets for each strain.

### *De novo* assembly

The SSAKE *de novo* assembler [171] was used to join the short single-end Illumina reads into longer blocks of continuous sequence, or contigs. Each of the four FASTQ files generated above was assembled by SSAKE under two independent conditions. First the default settings of SSAKE were used. Then the trim option was applied to each of the four input files during assembly, to trim two bases from the end of each contig once all possible other joins had been exhausted. This produced a total of eight SSAKE assemblies for each viral strain. These eight alternative sets of SSAKE contigs were combined and used as inputs to a long-read assembler, based on an approach used successfully for HTS assembly of HCMV genomes [137].

The Staden DNA sequence analysis package was used for further genome assembly of the long sequence contigs generated by SSAKE [172], [173]. The Pregap function was used to process and rename all contigs, which were then assembled using the standard “independent assembly” function of Gap4, with default settings. Contigs were sorted into descending size order and outputted as a normal consensus. This generated a multi-line FASTA formatted file that we inputted to NCBI's blast2seq program [174], for comparison to the PRV mosaic reference genome (Accession number NC_006151) [30]. This program produced pairwise alignments of each contig against the reference genome, allowing us to order the contigs along the genome and to flag potential bad joins generated by the assemblers. Contigs with suspicious joins were visually inspected in the Gap4 Contig Editor. These joins often occurred at extended runs of Gs or Cs, where disparate regions of the genome were joined solely as a result of overlapping mono-nucleotide stretches. The final assembly was created in gap4 by manually joining the minimum possible number of contigs. Final genome assemblies were further improved by PCR validation and repeat expansion, and verified by RFLP analysis (see below). All genome sequences are deposited with annotations (described below) in the NCBI Nucleotide (GenBank) collection: PRV Bartha: JF797217, PRV Kaplan: JF797218, PRV Becker: JF797219.

### Annotation of genes and coding sequences

Annotation of the new PRV genome sequences was created by BLAST homology-based transfer of annotations from the prior mosaic reference genome (NC_006151) to PRV Kaplan, using previously described scripts [81], [174]. Annotations of PRV Kaplan were then similarly transferred to PRV Becker and Bartha. Scripts for automated annotation transfer are available for download at http://genomics-pubs.princeton.edu/prv. Annotation transfer can fail when several base pairs of divergence or indels occur at the gene boundaries; these instances were addressed by manually varying the BLAST parameters to improve alignment and/or visually inspecting a pairwise alignment of the new strain against the reference. Entrez Gene IDs for all PRV, HSV-1 and VZV genes are listed in text format in Text S1, as well as hyperlinked in Table S6.

### Sequence alignment

The completed PRV genomes were aligned using the mVista genomics analysis tool with global LAGAN alignment [175], [176]. The VISTA Browser was used to visualize genome-wide conservation based on this alignment. The VZV genome (NC_001348) was used as an outgroup for tree generation in MacVector v11.1.2 (MacVector, Inc.) by the neighbor-joining method. One thousand rounds of bootstrap analysis provided confidence values for the branch points. Similar trees were obtained using alternative methods, such as clustering by the unweighted pair-group method with arithmetic mean (UPGMA) or following the precedent of single-gene comparison of the variable gC (UL44) nucleotide sequence [33], [34], [177].

### RFLP and Southern Blot analysis

Digestion of nucleocapsid DNA was performed to verify predicted fragment sizes corresponding to the newly assembled genomes. RFLP reactions utilized 4 µg nucleocapsid DNA per reaction, while Southern Blot digests used 1 µg nucleocapsid DNA. Reactions included viral nucleocapsid DNA, BamHI or SalI High Fidelity restriction enzymes (New England BioLabs), and supplied buffers and reagents as directed by the manufacturer; these were incubated at 37°C overnight. The addition of 5 µg/ml of ethidium bromide to an 0.8% agarose gel and to the 1X TAE running buffer allowed for enhanced UV visualization of fragments. Gel electrophoresis of the digested samples ran at 30 volts for approximately 48 hours at 4°C.

Southern blotting used the NEB Phototope-Star detection kit for nucleic acids (New England BioLabs) according to manufacturer's instructions. Briefly, the SalI RFLP gel was transferred to a nylon membrane and UV crosslinked. After blocking, the boiled probe was hybridized to the membrane overnight at 68°C, and detected by sequential application of streptavidin, biotinylated alkaline phosphatase, and finally the chemiluminescent reagent CDP-Star (New England BioLabs). The biotinylated probe was synthesized and HPLC-purified (Integrated DNA Technologies/IDT) to match SSR_Ka15795_ and the homologous SSRs in other strains. The probe consisted of three tandem copies of the SSR unit (a 15 mer), using the reverse-strand sequence of the SSR to allow for the incorporation of a biotinylated thymidine (T*, one per oligonucleotide): 5′-TCTCCCCTCCGTCCCTCTCCCCT*CCGTCCCTCTCCCCTCCGTCCC-3′.

### PCR validation of selected regions

Primers were designed for the amplification of several genes from nucleocapsid genomic DNA of all three PRV strains and their parental lysate DNA. Primer pairs are listed in Table S4 (in Text S1). To allow for easier PCR access, template DNA was boiled for 5 minutes and immediately cooled on ice. Initial PCRs were executed in 50 µl volumes using 1 µl of template. The reaction setup contained 1X Advantage 2 DNA polymerase (Clontech), 1X buffer as supplied by the manufacturer, 2% dimethyl sulfoxide, 1.2 M betaine (Sigma), each primer at a concentration of 0.5 µM, and each deoxynucleoside triphosphate at a concentration of 250 µM. Initial PCR conditions using an Eppendorf thermocycler are as follows: Initial denaturation at 95°C for 3 minutes, followed by 25 cycles of denaturation at 95°C for 30 seconds, primer annealing at 50°C for 30 seconds, and primer extension at 68°C for 2 minutes, with a final extension step at 68°C for 10 minutes. For more difficult gene amplifications an alternate reaction setup was used: 0.6 U Takara Ex Taq polymerase (Takara); 1X buffer as supplied by the manufacturer; 5% dimethyl sulfoxide; each primer at a concentration of 1 µM; each deoxynucleoside triphosphate, with equal amounts of dGTP and 7-deaza-2′-dGTP (Sigma Aldrich), at a concentration of 200 µM; and 1 µl of template DNA for a total reaction volume of 25 µl. Alternate PCR conditions were also used: Initial denaturation at 95°C for 5 minutes, followed by 40 cycles of denaturation at 95°C for 1 minute, gradient primer annealing temperatures from 55–75°C for 1 minute, and primer extension at 72°C for 2 minutes, with a final extension step at 72°C for 7 minutes.

For PCR validations of PRV Becker and Bartha parental DNA, we used lysates from the oldest available laboratory stocks of each virus. HTS data had already revealed that the oldest available stock of PRV Kaplan in the lab contained several hundred polymorphic base calls (described in Results and Figure 7C), so we instead compared results from PCR amplification of a stock of gH-null PRV Kaplan provided by Mettenleiter and colleagues [178]. By selecting these stocks, all of which were historically separated from the sequenced strains by multiple passages, we aimed to maximize the opportunity to detect sequence divergence relative to the new genomes.

### Western blot analysis

Cell lysates from PK15 cells were collected at 12 and 24 hours post infection into ice cold PBS and centrifuged for 3 minutes to pellet the cells and allow aspiration of the supernatant. The cells were lysed with RIPA light buffer (50 mM Tris/HCl (pH 8.0), 150 mM NaCl, 5 mM EDTA, 1% NP-40, 0.1% SDS, 0.1% Triton X-100). Insoluble cell debris was pelleted by centrifugation at 4°C, and the supernatant was collected for protein measurement. 50 µg of protein from the RIPA supernatant was brought up to a common volume using Laemli buffer (100 mM Tris/HCl (pH 6.8), 4% SDS, 200 mM DTT, 0.2% bromophenol blue, 20% glycerol) for each sample. These were boiled for 5 minutes at 95°C, electrophoresed through a 10% SDS-PAGE gel, and transferred to a nitrocellulose membrane (Whatman PROTRAN) using a Bio-Rad semi-dry transfer cell. The membranes were blocked using 5% non-fat milk and PBS-T. Primary and secondary antibodies were diluted in 1% non-fat milk in PBS-T. Proteins were visualized using rabbit polyclonal antibodies for gH (UL22) (1:2000) and VP1/2 (UL36) (1:10,000); mouse monoclonal antibodies for gB (UL27) (1:1000), VP5 (UL19) (1:1000) and β-actin (1:1000); goat horseradish peroxidase-conjugated secondary antibodies; and SuperSignal chemiluminescence reagents (Thermo Scientific) as indicated by the manufacturer's instructions. Band intensities were measured using the ImageJ (NIH) Gel Analyzer module.

### Coverage depth and polymorphic base detection

For quality control assessment of the finished genome assemblies, we used the Bowtie [170] and Samtools [179] software packages to assess the depth of sequence coverage and check for variant base calls. First, Bowtie (option –best) was used to align the Illumina sequence reads used for assembly against the finished genomes. Then three Samtools commands (view, sort, and pileup, with default options) were used to format the Bowtie alignment output and measure the depth of sequence read coverage (a pileup file) at each base of the finished genome sequence. The Integrated Genome Browser (IGB, [180]) was used to visualize each pileup graphically (a wiggle or wig plot; Figure S1 in Text S1). Finally, the Samtools varFilter command (default options, depth 40,000) was used to detect any variant base calls in the alignment of sequence reads back to the finished genomes. Assessment of polymorphic bases in the passaged (Becker p10) and non-purified (Kaplan n.p.) genomes was done by aligning sequence data for these stocks against the finished genome from the matching plaque-purified stock (i.e. Beckerp10 was aligned to the finished PRV Becker genome, and Kaplan n.p. to the PRV Kaplan genome).

Additional filtering was used to remove potential erroneous SNP calls [181]. These filters were based on a manual examination of all SNPs in strains Kaplan and Bartha. First, SNP locations were screened and flagged if they met any of the following criteria: adjacent to homopolymers of length ≥6, directional strand bias >85%, or overall coverage depth <100. All flagged SNPs were manually examined using the Integrative Genomics Viewer (IGV) to display sequence reads aligned to the genome sequence [182]. SNPs with likely homopolymer-based alignment error, unidirectional sequence read support, or signs of site-specific error were discarded [181]. Both filtered and unfiltered lists of DNA polymorphisms are available for download at http://genomics-pubs.princeton.edu/prv. Frequency distributions of polymorphic base calls were plotted using Prism v5.0 (GraphPad Software, Inc.).

### Estimation of G/C coverage bias

To measure G/C coverage bias, we followed the method of Frazer and colleagues [59] (Figure S2A in Text S1). Briefly, each genome was divided into sequential 10-mers. The coverage depth of each 10-mer was determined by taking the average coverage depth of the bases in the 10-mer. These were placed into bins according to G/C content, i.e. the number of G or C bases in the 10-mer. We recorded the number of 10-mers and the median coverage depth in each bin.

### Coverage Adjusted Perfect Repeat Expansion (CAPRE)

We used the coincidence of very high sequence coverage at perfect repeats in each PRV genome to estimate the actual length of these SSRs. The CAPRE script was applied only to selected regions meeting these criteria: an intergenic region, with coverage more than two standard deviations from the median, and centered on a perfect SSR with repeating units exceeding the median length of the filtered Illumina sequence reads. For each intergenic region meeting these criteria, an SSR unit that most closely matched the median Illumina read length was defined, and its genome position boundaries noted. The CAPRE script first determined the G/C content of the inputted SSR unit and used the G/C coverage bins above to obtain the expected median coverage depth for this SSR unit. The script then took the defined SSR unit boundaries and measured their observed sequence coverage. The script then estimated how many copies of the defined SSR unit would be needed to achieve the expected coverage depth, and inserted the appropriate number of SSR units into the genome sequence. The position of subsequent CAPRE regions was iteratively adjusted to account for expansion of the preceding region. To produce upper and lower estimates of SSR length, we ran the CAPRE script again and estimated the SSR length according to the upper and lower quartiles of observed sequence coverage (Figure S2 in Text S1) for each G/C content, instead of the median.

### Short Sequence Repeats (SSRs) comparison between strains

The location of SSRs throughout the PRV genome was mapped using MsatFinder and Tandem Repeat Finder (TRF) [183], [184]. MsatFinder detects perfect tandem repeats from homopolymers (1 repeating base) to hexamers (6 bases long). We searched for homopolymers of at least 6 bases long, and the following minimum number of repeating units for larger microsatellites: 5 units for di-, 4 units for tri-, and 3 units for quadri- to hexa-mers. TRF finds larger repeating units, and was designed to detect imperfect repeats that include minor base variations and indels. We ran TRF v4.04 with the following parameters: match 2, mismatch 5, delta 5, PM 80, PI 10, minScore 40, and maxPeriod 500. TRF output was pruned to remove overlapping repeats, preserving the SSR with higher alignment score. We utilized only TRF output with an alignment score of at least 40. This value is commonly used for other genome analyses, and we validated this cutoff for PRV by analyzing the number of repeats that would occur by chance in a shuffled version of the PRV Kaplan genome. Analysis of this shuffled genome detected 73 TRF SSRs in the randomized genome, vs. 637 in the PRV Kaplan genome. Thus approximately 1 out of every 10 TRF repeats might occur by chance, due to nucleotide composition.

Mapping and comparison of homologous SSRs on related PRV genomes were done by previously described methods [49], [125]. Briefly, we first aligned the complete PRV genomes using the mVista genomics analysis tool (LAGAN alignment option) [175], [176]. Sections of this alignment containing SSRs, as mapped in the PRV Kaplan genome, were screened for comparable SSRs in the orthologous regions of the Becker and Bartha genomes. This process was repeated using the lists of SSRs found in the PRV Becker and Bartha genomes. Screening of SSRs using all three genomes as a starting point allowed detection of SSRs that do not occur in all three strains, whose length or purity of repeating units is below threshold in PRV Kaplan but detectable in Becker or Bartha, or whose sequence is divergent enough to be scored as a separate SSR. Table S7 contains a full list of SSRs found in these three genomes. The identifier for each SSR denotes the genome from which its mapping was derived, as well as its starting position in that genome (*e.g.* SSR_Ka151_).

## Supporting Information

Text S1 This PDF file provides supporting tables, figures, legends, and text. **Tables S1–S5** include additional data described in the text. Legends describe data in the accompanying Figures S1–S5. Supplementary text lists accession numbers (Entrez Gene IDs) for all genes.
(PDF)

Table S6 This Excel file displays the percent of inter-strain protein variation in PRV, HSV-1, and VZV, along with hyperlinked Entrez Gene IDs and protein names.
(XLS)

Table S7 This Excel file provides a comprehensive list of SSRs detected in PRV Kaplan, Becker, and Bartha, with accompanying data on their location and characteristics.
(XLS)
