# Supplementary material for: A Wide Extent of Inter-Strain Diversity in Virulent and Vaccine Strains of Alphaherpesviruses
Source: PLoS Pathog. 2011 Oct 13;7(10):e1002282. doi: 10.1371/journal.ppat.1002282 (PMC3192842; doi:10.1371/journal.ppat.1002282)
Supplement: Text S1 — This PDF file provides supporting tables, figures, legends, and text. Tables S1–S5 include additional data described in the text. Legends describe data in the accompanying Figures S1–S5. Supplementary text lists accession numbers (Entrez Gene IDs) for all genes. (PDF) [file ppat.1002282.s001.pdf]

TEXT S1

SUPPLEMENTARY TABLES

Table S1. Summary of Illumina sequence reads generated for each PRV strain

Virus, Strain	Length (bp)	# Illumina sequence reads generated	Read length (# lanes)	Percent host DNA	Median sequence depth[a]
PRV Kaplan	140,377	16,780,084	75 bp (1)	8.6%	3,704
PRV Becker	141,113	16,063,614	51 & 75 bp (2)	6.4%	4,145
PRV Bartha	137,764	15,148,388	51 & 75 bp (2)	7.2%	4,137
Kaplan n.p. [b]	140,377	13,310,145	75 bp (2)	8.4%	3,285
Becker p10 [c]	141,113	9,272,515	75 bp (1)	7.3%	2,342

[a] Depth of sequence coverage at each base, when sequence reads were aligned against the finished genome assembly.
[b] n.p., not purified.
[c] p10, passage 10.

Table S2. PCR validations of sections of PRV Kaplan, Becker, and Bartha genomes

PRV Strain	Target gene	PCR validated region	Differences from reference or mosaic
Becker	UL3.5 [P]	92,058 - 92,666	deletion & AA changes
Becker	VP1/2 (UL36) [P]	(a) 32,801 - 33,952	deletions & insertions
		(b) 34,530 - 35,945	
		(c) 40,950 - 41,637	
Becker	VP13/14 (UL47)	11,467 - 12,207	deletion & AA changes
Becker	VP22 (UL49) [P]	9,173 - 9,586	insertions, deletions & AA changes
Becker	Left edge of IR; right edge of TR	100,806 - 101,112<br>140,746 - 141,052	SSR divergence
Becker	ICP22 (US1) [P]	115,046 - 115,797	deletions & insertions
Becker	5'UTR of ICP22 (US1)	114,499 - 114,841	homopolymer run; low coverage
Becker	gG (US4)	117,445 - 118,501	frameshift in PRV Rice strain
Bartha	VP1/2 (UL36) [P]	(a) 32,245 - 33,583	deletions, insertions & additional repeat unit
		(b) 34,500 - 35,569	
Bartha	UL43 [P]	52,850 - 53,500	frameshift & AA changes
Bartha	VP13/14 (UL47)	11,324 - 12,378	deletion & AA changes
Bartha	Left edge of IR; right edge of TR	107,280 - 107,856<br>129,992 - 130,568	SSR divergence
Bartha	5'UTR of ICP22 (US1)	114,341 - 114,797	homopolymer run; low coverage
Bartha	ICP22 (US1) [P]	115,270 - 115,879	deletions & insertions
Bartha	gG (US4)	118,417 - 119,137	frameshift in PRV Rice strain
Kaplan	gG (US4)	118,021 - 118,575	frameshift in PRV Rice strain
Kaplan	UL43 [P]	52,135 - 53,019	AA changes
Kaplan	gC (UL44) [P]	53,032 - 53,689	AA changes
		53,804 - 54,272	

[P] These PCRs were also validated by amplifying from a parental virus stock and comparing the result to the sequence of the plaque-purified isolates. No base pair differences, indels, or other changes were detected in these parental PCRs.

Table S3. Selected SSRs with length estimated by CAPRE, in each PRV genome

Strain where CAPRE was applied	SSR ID (see also Table S7)	# SSR units by *de novo* assembly	# SSR units by CAPRE (lower-upper estimates) [a]	Location (length); see also Figure S1 (in Text S1)
PRV Kaplan	SSR_{Ka2093}	4.3	8.3 (6.3-12.3)	Between ORF-1 & UL54 (21mer)
PRV Kaplan	$SSR_{Ka15795}$	4.7	13.7 (10.7-22.7)	Between UL46 & UL27 (15mer)
PRV Kaplan	$SSR_{Ka107138}$	4.5	12.5 (10.5-18.5)	Between IE180 & OriS (in IR) (19mer)
PRV Becker	SSR_{Be152}	3	5 (4-7)	Left terminus (34mer)
PRV Becker	$SSR_{Be15739}$	8	59 (41-92)	Between UL46 & UL27 (15mer)
PRV Becker	$SSR_{Be115911}$	9.8	20.6 (16.6-26.6)	Between US1 & edge of IR (19mer)
PRV Bartha	$SSR_{Ba15751}$	6.4	18.4 (15.4-27.4)	Between UL46 & UL27 (15mer)
PRV Bartha	$SSR_{Ba62427}$	7.5	11.5 (7.5-11.5)	Between US22 and OriL (11mer)
PRV Bartha	$SSR_{Ba79489}$	6.1	8.1 (6.9-12.9)	Between UL11 & UL10 (12mer)
PRV Bartha	$SSR_{Ba100233}$	6.7	8.7 (8.7-10.7)	Between left edge IR & IE180 (22mer)

[a] CAPRE estimates of SSR length are based on the median expected depth of coverage for a given G/C content, with upper and lower estimates based on the upper and lower quartiles of the coverage range for that G/C content (Figure S2 in Text S1).

Table S4. Primers used for PCR on PRV Kaplan, Becker, and Bartha genomes

Gene	Forward Primer	Reverse Primer	Strain
UL3.5	CTGTACATCGTCGTGCTCGT	AGATGTTTATCCTGTGCCGC	Becker
VP1/2 (UL36), (a)	AGTCCCACAAGTTCCCCAAT	ATCAACCTGCGGGACATCT	Becker
VP1/2 (UL36), (b)	GTGTGTTCGCACTTGTCTGC	CTCCATCATCATGGGCGAC	Becker
VP1/2 (UL36), (c)	ACCACCGTCTGGTCGTAGA	ATCAGTATGACCCCGACCTG	Becker
UL47*	GTGGTCGGGGCGTATAAAG	AGGAACATGGCGTGGTGCAG	Becker
VP22 (UL49)	TCTCTTCCATCGTCTCCACC	ATACACTTTTCCCTTCCGCC	Becker
ICP22 (US1)	CGCCCTCTTTGACTTTTACG	AGAGAGTTGAGGTTCGAGCG	Becker
IR-TR	GGCTAATTTGCATAAATTTGCATACA	GCGATATGCAGATGAGATCCGT	Becker
gG (US4)	CTGTACATCGTCGTGCTCGT	GCCGTACGCAAATTAGGTGT	Becker
ICP22 (US1) 5' UTR*	TCGTGGCTCACGCTGACGTCATGCG	GGCCCGTACTCGGGGTGCTCGACG	Becker & Bartha
VP1/2 (UL36), (a)*	AGTCCCACAAGTTCCCCAAT	AAGCCGCCTCATCCTCTTCT	Bartha
VP1/2 (UL36), (b)*	TCACCATCGGATTTCTTGG	ACGACAACCCCATCGAGAAC	Bartha
UL43	GGTGTATAAAAGAGAGGTCGCA	CATGATGACCAGCACGATG	Bartha
VP13/14 (UL47)*	ATGTTTGAGATGTGACGCCG	AGGAACATGGCGTGGTGCAG	Bartha
ICP22 (US1)	CGCCCTCTTTGACTTTTACG	AGAGAGTTGAGGTTCGAGCG	Bartha
IR-TR*	CAATCAGATGATTTTCGGGG	GCGACAAAAGTATCCAAGGC	Bartha
gG (US4)	ATCGTCGTGCTCGTCTTTG	GTAGATCGAGGTGCGGATGT	Bartha
UL43*	CCACGCCTACGGAGATTTAT	TCGTCAAAGTACTCGGGGTC	Kaplan
gC (UL44)	AGTGGATGGTCTCCCAGATG	CGGACTCGCTGTCGTTTATT	Kaplan
gG (US4)	CTGTACATCGTCGTGCTCGT	GTAGATCGAGGTGCGGATGT	Kaplan

* Indicates genes for which alternative PCR reaction setup was used.

Table S5. Protein-coding differences between PRV Kaplan and the PRV mosaic genome NC_006151 show that most differences are due to non-Kaplan source strains

Protein	Amino acid differences, relative to PRV mosaic protein sequence [a]	Notes
VP16 (UL48)	186-187(PW>RL)	
gB (UL27)	71(+VPG), 72-73(GT>SP), A75L, S93T, T386I, S442N, A493S, A553T, N679S	Mosaic source: Becker
ICP18.5 (UL28)	T58M, H225R, 253-258(ASTAAA>Δ), V561L, R563H, G694A	Mosaic source: Becker
ICP8 (UL29)	E24A, V155A, P223A, P230A, A327S, A344G, A346G, 348-349(SA>AG), 394-395(AR>GGG), L428P, 519-525(SRASARR>LPRFGAPT), T592A, V1144A	Includes frameshift; Mosaic source: TNL & Becker
UL30	G939R	
VP1/2 (UL36)	S184G, P1429R, 2791-2802(PPSRPAAPPPAP>Δ)	
RR2 (UL40)	P44A	
vhs (UL41)	P277R	
UL43	F239L, V279L, T299A, A311V, 328-336(GAARGGPRP>ALLEADHVR)	Includes frameshift; Mosaic source: Becker
gC (UL44)	D30G, 68-69(PV>RA), K99E, A179T, V181A, 183(V>ED), E186G, A316D	Mosaic source: Becker
UL25	A235P, P277A	
TK (UL23)	T293A	Mosaic source: NIA-3
VP23 (UL18)	54(D>AY)	
UL15	C42R	
VP18.8 (UL13)	F233V	
UL8	P216L	
UL7	T121N	
ICP0 (EP0)	33-34(LT>QS), N42K, T172A	Mosaic source: Indiana-Funkhauser
ICP4 (IE180)	25(+A), 1033(+GAA), V1177A	
gG (US4)	V244A, S249L, A296V, 299-301(PAT>AGDG), A307D, 330-374(RPPASSPPPPPPRPHPRGRDHDHDHGHHRADDRGPQRHHRLPPEPT >AAARFFAASTTPRAPTRGAEITTMTTVTTVRTTEDPSGITDCRRSD), I483T	Includes frameshift; Mosaic source: Rice
gI (US7)	P207Q, I210L, V249A, A255V, G257D, L261-, G282S	Mosaic source: Becker
gE (US8)	M124T, Q162R, R180Q, A299T, A446G, D499V	Mosaic source: Becker
US9	A24P	Mosaic source: Becker
US2	T52S, I130L, I146V	Mosaic source: NIA-3

[a] Single AA residues changes are written in standard format, including the mosaic strain AA, its position, and the AA residue found in the PRV Kaplan strain reported here, *e.g.* S100P. Insertions (relative to the mosaic sequence) are indicated by the AA position in the mosaic sequence, followed by "+" and the new AAs, *e.g.* 100(+RR). Deletions are indicated by the symbol Δ. Sequential changes are combined and shown with the AA positions first, followed by the relevant mosaic sequence AA residues, then ">", and finally the new Kaplan strain AA residues, *e.g.* 100-102(RAR>EDA).

Two Supplementary Tables are separately attached as Excel files. Title & description of each are here.

Table S6. Percent of inter-strain protein variation in PRV, HSV-1, and VZV

This table includes all data used to generate Figure 5, as well as hyperlinked NCBI Accession numbers for all individual genes of PRV, HSV-1, and VZV. The first column lists the common protein name for each gene in the alpha-herpesvirus genome, followed by five columns each for PRV, HSV-1, and VZV: gene name (which varies across viruses), amino acid (AA) length, total # AA differences across *x* number of strains (3 for PRV, 3 for HSV-1, 18 for VZV), percent AA variation across *x* number of strains, and web-linked NCBI accession ID. Genes in the PRV Bartha deletion region are noted, along with those not found across all three virus species. Data for HSV-1 and VZV inter-strain variation are derived from Szpara *et al.* (2010) [81] and Tyler *et al.* (2007) [78,79].

Table S7. Comprehensive list of SSRs detected in PRV Kaplan, Becker, and Bartha.

This table includes all data on SSR locations and characteristics in the PRV Kaplan, Becker, and Bartha strain genomes, although with comparable data on the prior mosaic reference genome (NC_006151). A separate worksheet tab within the excel file contains data for each strain. Columns include SSR ID (including strain and start position), start and end positions on the genome, repeat unit length, SSR purity (percent match), percent indels (insertions or deletions), TRF alignment score, VarScore, consensus sequence of the repeat unit, full sequence of the SSR, number of repeat units found in each genome (Kaplan, Becker, Bartha, and the mosaic reference; NULL or NC if not found), genomic region (*e.g.* intergenic, promoter, or coding), gene name (if relevant), and SSR search ID (“msat” indicates an SSR found by MsatFinder, and “trf” indicates an SSR found by Tandem Repeat Finder).

Supplementary Figure 1

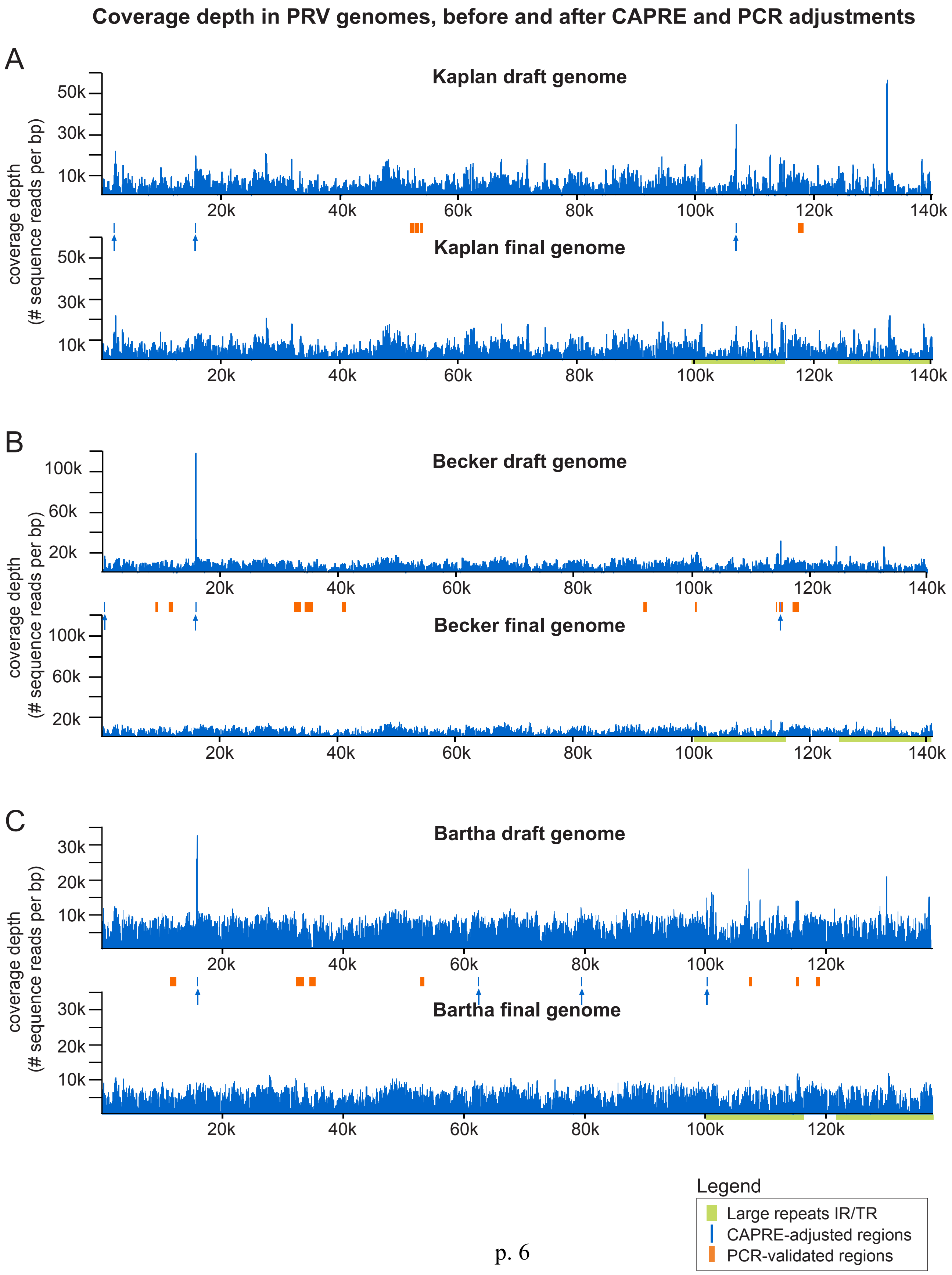

Supplementary Figure 1 Legend

Figure S1. Illumina sequencing coverage depth of PRV strain genomes, before and after CAPRE and PCR adjustments. Line graphs depict depth of Illumina sequence read coverage per base of the PRV genome, for strains Kaplan (A), Becker (B), and Bartha (C). The initial draft assembly of each genome (top half of each panel) revealed one or more sites of very high sequence coverage (>2 standard deviations above the median), centered over perfect SSRs in intergenic regions. Coverage-Adjusted Perfect Repeat Expansion (CAPRE) estimation of the actual width of these perfect SSRs provided a more even depth of sequence coverage in the finished genomes (bottom half of each panel). PCR validation of several genome regions improved the assembly further. Blue lines located between the draft and final genome panels, whose location is highlighted by arrows, indicate CAPRE-estimated regions; orange bars highlight PCR-verified regions. Position on the genome is represented by x-axis numbering on all graphs, and the large IR/TR regions (green boxes) are highlighted on each final genome assembly for orientation purposes. Further detail of ORF locations is found in Figure 1.

Supplementary Figure 2

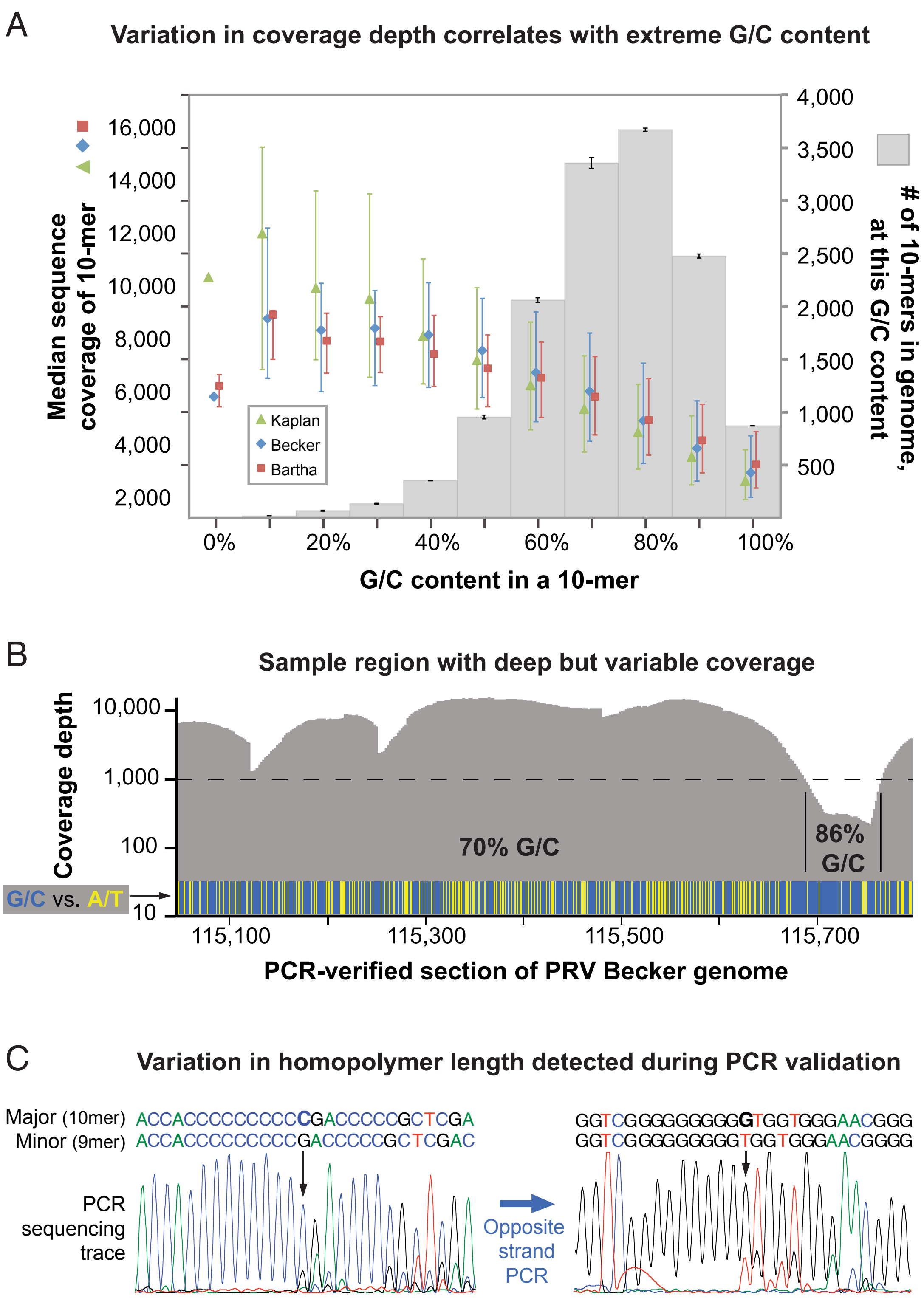

Supplementary Figure 2 Legend

Figure S2. Variation in coverage depth and homopolymer length in the PRV genome. A) On the left y-axis, consecutive 10-mers across each PRV strain genome were analyzed for their G/C content, as well as their median depth of Illumina sequence coverage. Colored points and error bars display the median and standard deviation per bin, for each genome. Gray histogram bars and the right y-axis summarize the total number of 10-mers in each G/C-content bin. The G/C-rich PRV genome has very few 10-mers with 0% G/C content (far left) and many with higher G/C contents (far right). B) Sample of variation in coverage depth from a section of PRV Becker near the ICP22 (US1) gene. Depths vary from 14,000 sequence reads/base to as little as 250 sequence reads/base within 1 kb (y-axis plotted on log10 scale). The DNA sequence of this region has been color-coded to demonstrate G/C (blue) versus A/T (yellow) content of the forward strand. A subset of this area with coverage depth of <1000 reads/base correlates with higher G/C content. This region was additionally validated by PCR sequencing, confirming that the coverage depth variation is not due to an assembly error. C) PCR verification of the non-coding region upstream of PRV Becker US1 detected variation in length of a C10 homopolymer. A minority of the PCR products amplified from this plaque-purified stock appear to have a C9 homopolymer, which is also visible as a G9 minority variant upon sequencing of the reverse strand.

Supplementary Figure 3

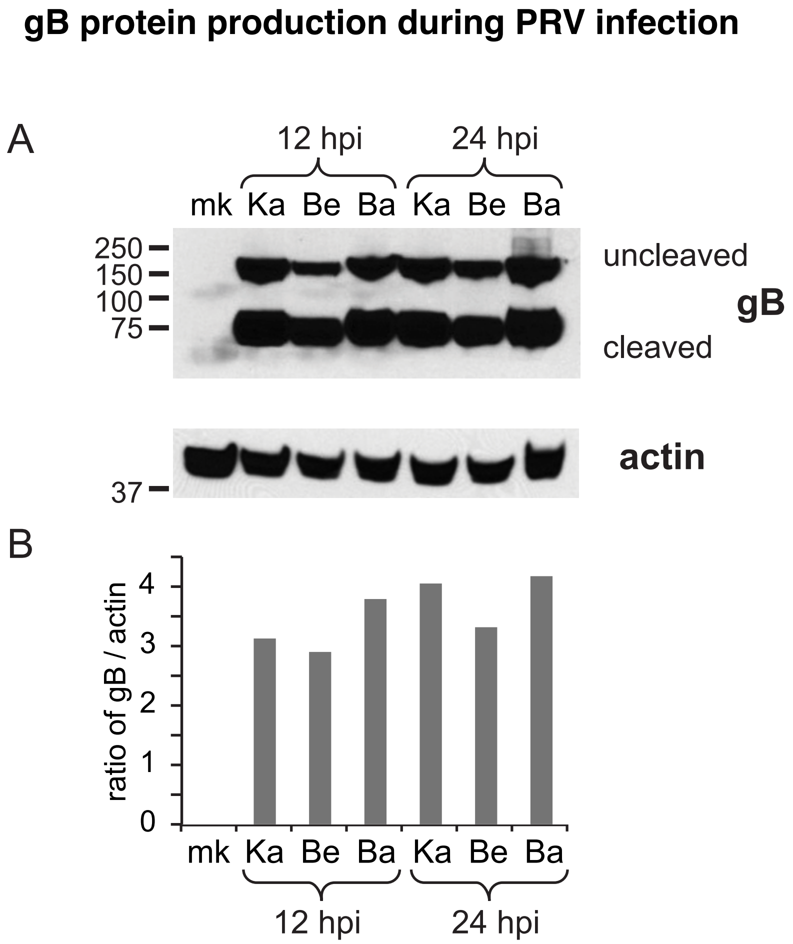

Figure S3. Inter-strain variation in protein levels of gB. A) Western blot analysis of infected cell lysates demonstrates that PRV Bartha produces cleaved gB (UL27) despite several AA residue changes adjacent to its furin cleavage site. Levels of cellular actin are shown for comparison and as a loading control. B) Ratio of gB vs. actin in each sample, using the ImageJ Gel Analyzer module. Equivalent amounts of protein were loaded in each lane. The blot was cut, with gB measured on the upper half and actin on the lower half to demonstrate equal numbers of cells contributing to each lysate. The same lysates were used for the analyses in Figure 4; these are representative of three separate experiments. Positions of a standard marker are noted on the left.

Supplementary Figure 4

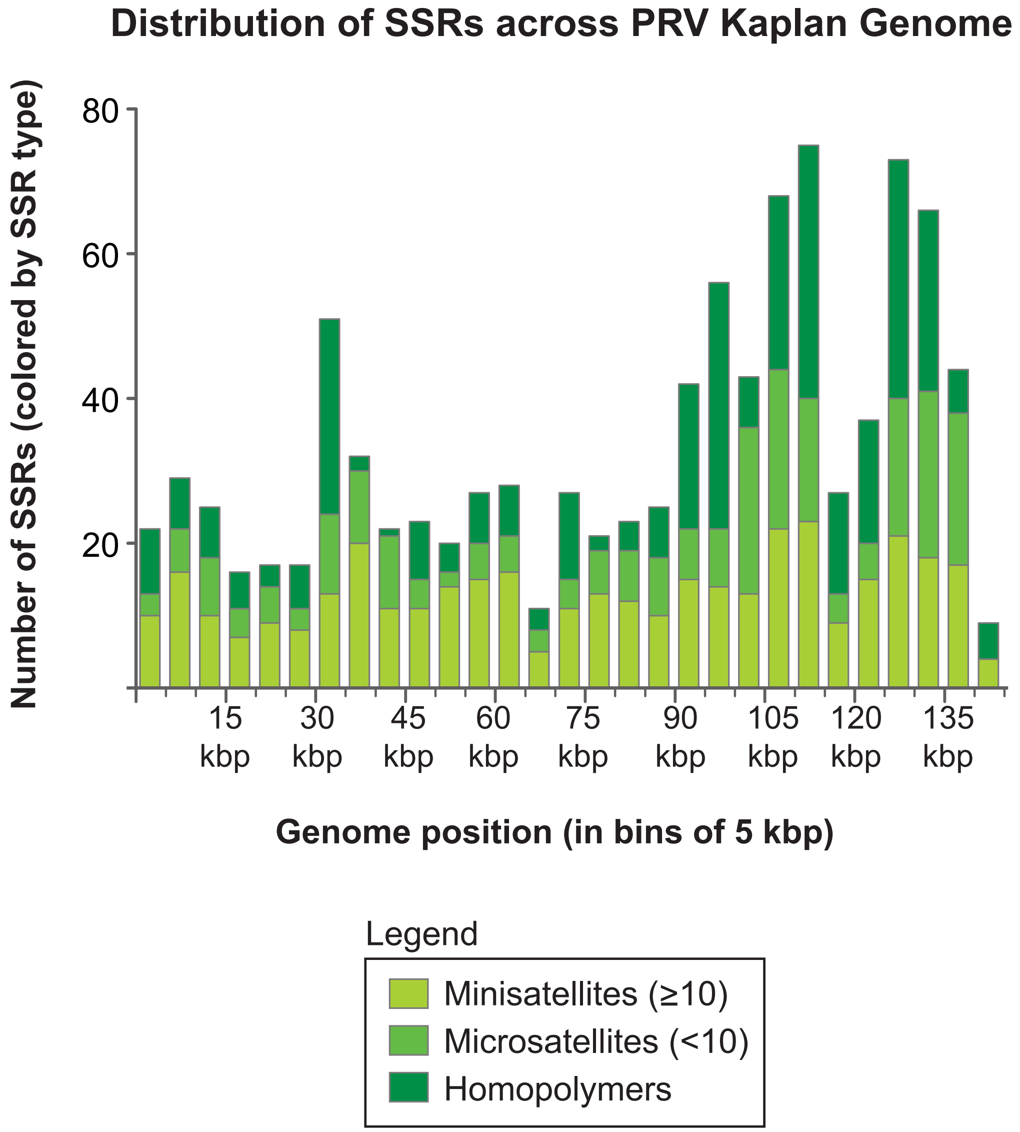

Figure S4. Distribution of SSR locations across the PRV Kaplan genome. SSRs in the PRV Kaplan genome were grouped in bins of 5 kb across the genome, and the number of SSRs whose start position fell into each bin was summed. Data is plotted as a cumulative histogram of the different SSR groups: homopolymers (length ≥ 6), microsatellites (<10 bp unit length), and minisatellites (≥10 bp unit length). More SSRs are found in the IR/TR and unique short (US) region (100 kb and higher on x-axis) than in the unique long (UL) region. See Figure 1 for further detail of ORF and SSR locations on the PRV genome.

Supplementary Figure 5

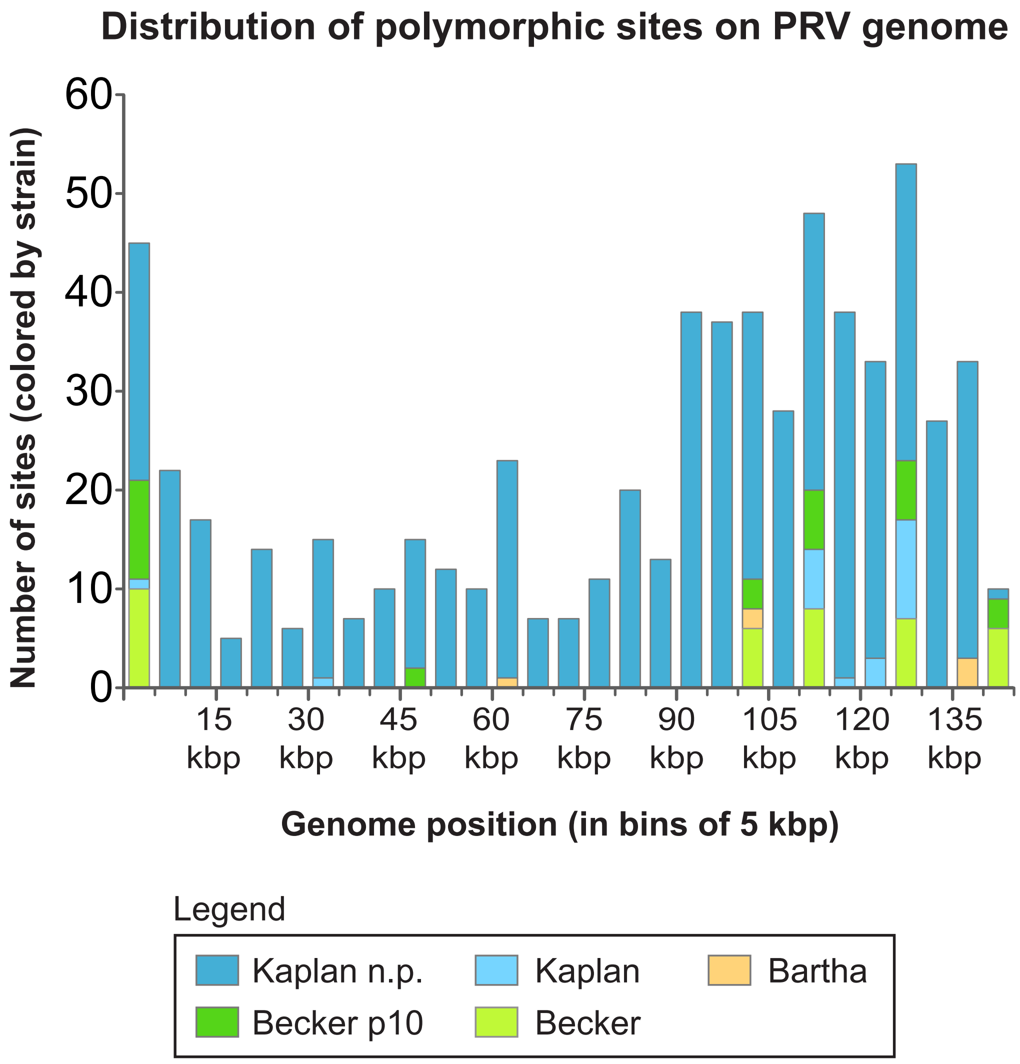

Figure S5. Distribution of polymorphic sites on the PRV genome. Polymorphic base calls in each PRV genome were binned by genome position (bins of 5 kb), and the sum of polymorphisms per bin plotted as a cumulative histogram. The unpurified historical Kaplan stock (Kaplan n.p., dark blue bars) displays the largest number of polymorphic sites, with more occurring in the IR/TR and US region (100 kb and higher on x-axis) than in the UL region. Although there are fewer polymorphic sites in all other strains, their distribution is similar. See Figure 1 for further detail of ORF locations on the PRV genome.

SUPPLEMENTARY TEXT

Accession Numbers for all Genes and Proteins

This paragraph provides NCBI GeneID accession numbers for all genes and proteins mentioned in the text (these are also included in Table S6). Genes are listed in order of occurrence along the PRV genome, as shown in Figures 1 and 3. Weblinked versions of the same information are also included in Table S6.

UL56 (ORF-1): PRV GeneID 2952514, HSV-1 GeneID 2703428, VZV GeneID 1487696; **ICP27 (UL54):** PRV GeneID 2952533, HSV-1 GeneID 2703426, VZV GeneID 1487672; **gK (UL53):** PRV GeneID 2952532, HSV-1 GeneID 2703425, VZV GeneID 1487673; **UL52:** PRV GeneID 2952515, HSV-1 GeneID 2703423, VZV GeneID 1487676; **UL51:** PRV GeneID 2952534, HSV-1 GeneID 2703422, VZV GeneID 1487677; **dUTPase (UL50):** PRV GeneID 2952537, HSV-1 GeneID 2703421, VZV GeneID 1487671; **gN (UL49.5):** PRV GeneID 2952538, HSV-1 GeneID 2703419, VZV GeneID 4711773; **VP22 (UL49):** PRV GeneID 2952503, HSV-1 GeneID 2703417, VZV GeneID 1487674; **VP16 (UL48):** PRV GeneID 2952504, HSV-1 GeneID 2703416, VZV GeneID 1487675; **VP13/14 (UL47):** PRV GeneID 2952518, HSV-1 GeneID 2703415, VZV GeneID 1487654; **VP11/12 (UL46):** PRV GeneID 2952519, HSV-1 GeneID 2703413, VZV GeneID 1487655; **gB (UL27):** PRV GeneID 2952558, HSV-1 GeneID 2703455, VZV GeneID 1487662; **ICP18.5 (UL28):** PRV GeneID 2952557, HSV-1 GeneID 2703457, VZV GeneID 1487661; **ICP8 (UL29):** PRV GeneID 2952495, HSV-1 GeneID 2703458, VZV GeneID 1487713; **UL30:** PRV GeneID 2952496, HSV-1 GeneID 2703462, VZV GeneID 1487712; **UL31:** PRV GeneID 2952498, HSV-1 GeneID 2703350, VZV GeneID 1487666; **UL32:** PRV GeneID 2952497, HSV-1 GeneID 2703352, VZV GeneID 1487694; **UL33:** PRV GeneID 2952545, HSV-1 GeneID 2703353, VZV GeneID 1487665; **UL34:** PRV GeneID 2952546, HSV-1 GeneID 2703355, VZV GeneID 1487693; **VP26 (UL35):** PRV GeneID 2952490, HSV-1 GeneID 2703356, VZV GeneID 1487705; **VP1/2 (UL36):** PRV GeneID 2952494, HSV-1 GeneID 2703357, VZV GeneID 1487704; **UL37:** PRV GeneID 2952491, HSV-1 GeneID 2703358, VZV GeneID 1487686; **VP19c (UL38):** PRV GeneID 2952492, HSV-1 GeneID 2703359, VZV GeneID 1487685; **RR1 (UL39):** PRV GeneID 2952535, HSV-1 GeneID 2703361, VZV GeneID 1487716; **RR2 (UL40):** PRV GeneID 2952536, HSV-1 GeneID 2703364, VZV GeneID 1487715; **vhs (UL41):** PRV GeneID 2952522, HSV-1 GeneID 2703365, VZV GeneID 1487714; **UL42:** PRV GeneID 2952523, HSV-1 GeneID 2703407, VZV GeneID 1487653; **UL43:** PRV GeneID 2952524, HSV-1 GeneID 2703408, VZV GeneID 1487652; **gC (UL44):** PRV GeneID 2952505, HSV-1 GeneID 2703410, VZV GeneID 1487660; **scaffold (UL26.5):** PRV GeneID 2952525, HSV-1 GeneID 2703454, VZV GeneID 4711772; **VP24 (UL26):** PRV GeneID 2952508, HSV-1 GeneID 2703453, VZV GeneID 1487717; **UL25:** PRV GeneID 2952507, HSV-1 GeneID 2703377, VZV GeneID 1487687; **UL24:** PRV GeneID 2952506, HSV-1 GeneID 2703375, VZV GeneID 1487688; **TK (UL23):** PRV GeneID 2952559, HSV-1 GeneID 2703374, VZV GeneID 1487667; **gH (UL22):** PRV GeneID 2952560, HSV-1 GeneID 2703373, VZV GeneID 1487668; UL21: PRV GeneID 2952541, HSV-1 GeneID 2703372, VZV GeneID 1487706; UL20: PRV GeneID 2952542, HSV-1 GeneID 2703371, VZV GeneID 1487707; **VP5 (UL19):** PRV GeneID 2952543, HSV-1 GeneID 2703368, VZV GeneID 1487708; **VP23 (UL18):** PRV GeneID 2952544, HSV-1 GeneID 2703366, VZV GeneID 1487669; **UL17:** PRV GeneID 2952555, HSV-1 GeneID 2703388, VZV GeneID 1487670; **UL16:** PRV GeneID 2952556, HSV-1 GeneID 2703386, VZV

GeneID 1487718; **UL15:** PRV GeneID 2952554, HSV-1 GeneID 2703385, VZV GeneID 1487719; **UL14:** PRV GeneID 2952529, HSV-1 GeneID 2703384, VZV GeneID 1487720; **VP18.8 (UL13):** PRV GeneID 2952530, HSV-1 GeneID 2703383, VZV GeneID 1487678; **AN (UL12):** PRV GeneID 2952531, HSV-1 GeneID 2703382, VZV GeneID 1487679; **UL11:** PRV GeneID 2952499, HSV-1 GeneID 2703380, VZV GeneID 1487656; **gM (UL10):** PRV GeneID 2952500, HSV-1 GeneID 2703379, VZV GeneID 1487658; **OBP (UL9):** PRV GeneID 2952547, HSV-1 GeneID 2703434, VZV GeneID 1487657; **UL8:** PRV GeneID 2952549, HSV-1 GeneID 2703432, VZV GeneID 1487721; **UL7:** PRV GeneID 2952509, HSV-1 GeneID 2703431, VZV GeneID 1487722; **UL6:** PRV GeneID 2952550, HSV-1 GeneID 2703429, VZV GeneID 1487723; **UL5:** PRV GeneID 2952510, HSV-1 GeneID 2703420, VZV GeneID 1487682; **UL4:** PRV GeneID 2952526, HSV-1 GeneID 2703362, VZV GeneID 1487683; **UL3:** PRV GeneID 2952501, HSV-1 GeneID 2703461, VZV GeneID 1487690; **UNG (UL2):** PRV GeneID 2952528, HSV-1 GeneID 2703370, VZV GeneID 1487691; **gL (UL1):** PRV GeneID 2952527, HSV-1 GeneID 2703393, VZV GeneID 1487692; **ICP0 (EP0):** PRV GeneID 2952512, HSV-1 GeneID 2703389, VZV GeneID 1487698; **ICP4 (IE180):** PRV GeneID 2952540, HSV-1 GeneID 2703392, VZV GeneID 1487695; **ICP22 (US1):** PRV GeneID 2952489, HSV-1 GeneID 2703435, VZV GeneID 1487700; **PK (US3):** PRV GeneID 2952561, HSV-1 GeneID 2703401, VZV GeneID 1487703; **gG (US4):** PRV GeneID 2952520, HSV-1 GeneID 2703404; **gD (US6):** PRV GeneID 2952521, HSV-1 GeneID 2703444; **gI (US7):** PRV GeneID 2952516, HSV-1 GeneID 2703446, VZV GeneID 1487689; **gE (US8):** PRV GeneID 2952517, HSV-1 GeneID 2703448, VZV GeneID 1487709; **US9:** PRV GeneID 2952552, HSV-1 GeneID 2703452, VZV GeneID 1487702; **US2:** PRV GeneID 2952553, HSV-1 GeneID 2703399; **UL3.5:** PRV GeneID 2952502, VZV GeneID 1487684; **ICP34.5 (RL1):** HSV-1 GeneID 2703395; UL45: HSV-1 GeneID 2703412; **UL55:** HSV-1 GeneID 2703427; **US10:** HSV-1 GeneID 2703436, VZV GeneID 1487701; **US11:** HSV-1 GeneID 2703439, VZV GeneID 1487681; **ICP47 (US12):** HSV-1 GeneID 2703441; **gJ (US5):** HSV-1 GeneID 2703406; **US8A:** HSV-1 GeneID 2703450; **ORF1:** VZV GeneID 1487664; **ORF2:** VZV GeneID 1487680; **TS (ORF13):** VZV GeneID 1487659; **ORF32:** VZV GeneID 1487663.
